# A data-driven and interpretable hybrid quantum-classical model for male infertility risk assessment

**DOI:** 10.3389/fmedt.2026.1853728

**Published:** 2026-10-02

**Authors:** T. Sarath, K. Brindha

**Affiliations:** School of Computer Science Engineering and Information Systems, Vellore Institute of Technology, Vellore, Tamil Nadu, India

**Keywords:** artificial intelligence, electronic health record, identifying disease, infertility, LSTM model, male, quantum computing

## Abstract

**Introduction:**

Nearly 15% of couples worldwide experience infertility, with Male Infertility (MI) accounting for 30–50% of these cases. Despite this burden, research on MI lags behind female infertility, particularly in understanding how demographic, clinical, lifestyle, and environmental factors interact to shape reproductive outcomes. Traditional approaches typically examine isolated indicators and struggle to capture the high-dimensional, nonlinear patterns present in Electronic Health Record (EHR) data. This study proposes a data-driven, interpretable framework integrating global fertility rate trends with EHR-derived male fertility data to (i) identify key determinants of MI, (ii) compare multiple classical Long Short-Term Memory (LSTM) architectures against a novel Quantum LSTM model embedding a Variational Quantum Circuit (VQC) directly within the LSTM gating mechanism, and (iii) evaluate the feasibility of quantum-enhanced sequential learning on small, heterogeneous healthcare datasets.

**Methods:**

Two public datasets were used: a global fertility rate time series (1960–2020) and an EHR–derived male fertility dataset (*n* = 100, 10 features). After preprocessing (scaling, encoding, multicollinearity checks, and class-imbalance correction), several classical LSTM variants and the proposed Quantum LSTM were trained and validated using 5-fold cross-validation, repeated random train–test splits, and bootstrap resampling. Performance was assessed via accuracy, precision, recall, F1-score, and computational time, with statistical significance evaluated using Friedman and Wilcoxon signed-rank tests. SHAP (SHapley Additive exPlanations) was applied for feature-level and model-level interpretability.

**Results:**

Among classical models, unilateral LSTM performed most consistently (91.2% accuracy on fertility-rate data; 92.1% on male-fertility data). The Quantum LSTM achieved the highest accuracy on both datasets (92.6% and 93.8% respectively), a modest but statistically significant improvement (Wilcoxon *p* = 0.031 and *p* = 0.024; Cohen's *d* = 0.42–0.48) with narrower bootstrap confidence intervals, indicating greater stability. SHAP analysis identified daily sitting duration and age as the most influential predictors of male fertility outcomes, followed by season and alcohol consumption frequency, with clinical history variables contributing comparatively less.

**Discussion:**

These findings demonstrate the feasibility of embedding quantum circuits within LSTM gating for small-scale healthcare prediction tasks, yielding incremental rather than transformative gains over classical architectures. Observed associations are exploratory and non-causal, and given the limited sample size (*n* = 100), single-center source, and simulator-based quantum execution, results should be interpreted as hypothesis-generating rather than clinically conclusive. This work establishes a reproducible benchmarking framework combining classical and quantum-enhanced LSTM models with SHAP-based explainability for early MI risk stratification; future work should validate findings on larger, multi-center cohorts and real quantum hardware.

## 1 Introduction

Globally, infertility affects over 15% of couples of reproductive age, with male infertility (MI) accounting for approximately 30%–50% of cases (1). Despite its substantial global burden and rising incidence, research on MI remains limited compared to female infertility, particularly in understanding how multiple lifestyle, environmental, medical, and physiological factors interact to influence reproductive outcomes. The complicated and diverse characteristics of infertility are better captured by modeling the cumulative multivariate effects; however, most existing research only considers single indicators, such as hormone levels, genetic mutations, alcohol use, or fever history. The absence of comprehensive data-driven exploration has hindered early diagnosis, risk assessment, and personalized clinical management in men.

In contrast, EHRs facilitate extensive research on MI. Hidden relationships may exist among the numerous demographic, clinical, lifestyle, and temporal data found in EHRs. Nevertheless, there are methodological obstacles associated with electronic health record (EHR) data owing to their high dimensionality, variability, and temporal interdependence (2). Often, the complex non-linear patterns associated with MI risk cannot be captured by traditional statistical or rule-based methods because these methods depend on established assumptions or have limited feature interactions.

AI, particularly long short-term memory (LSTM)-based deep learning (DL), provides a powerful technique for modeling intricate temporal and multivariate relationships. Recent progress in quantum computing (QC) has further demonstrated the potential of quantum-enhanced neural architectures to extract richer feature representations from small, noisy, or heterogeneous datasets. There is growing evidence supporting AI-driven quantum-enhanced models for predicting present and future health risks. Based on a literature study, health outcome predictions include an increased risk of diabetes, cardiovascular disease, autoimmune diseases, and chronic kidney disease. These studies used both retrospective and prospective case–control and cohort designs. Despite this, prior studies have not employed a data-driven strategy to catalogue the entire spectrum of comorbidities that pose risks to male reproductive health (3).

In light of these limitations, this study proposes a comprehensive, interpretable, and data-driven framework that integrates (i) global multidecade fertility rate analysis, (ii) EHR-derived male fertility data, and (iii) a comparative evaluation of multiple classical LSTM models alongside a proposed Quantum LSTM architecture. The objectives are the following:

1. Identify key medical and lifestyle factors influencing male fertility using multivariate EHR data.
2. Compare the prediction abilities of several LSTM models using small, diverse datasets.
3. Determine whether a Quantum LSTM model outperforms conventional approaches for fertility prediction.
4. Evaluate the feasibility of quantum-enhanced representation learning by investigating whether hybrid quantum-classical architectures provide incremental improvements over conventional LSTM models.
5. Improve clinical comprehension of male infertility risk factors by providing interpretable quantitative and visual insights.

The proposed Quantum LSTM architecture in this study differs from existing hybrid quantum-classical models in a key structural way: the variational quantum circuit (VQC) with angle encoding is integrated directly into the LSTM memory cell gating mechanism rather than being used as a postprocessing step. This allows quantum superposition and entanglement to directly influence temporal memory updates. The primary motivation for applying quantum computation to this dataset was not primarily accuracy gain but feasibility exploration and proof of concept for quantum-enhanced clinical AI pipelines. Unlike many existing hybrid quantum-classical models that utilize variational quantum circuits only as postprocessing feature extractors, the proposed Quantum LSTM incorporates the quantum circuit directly within the LSTM input gating mechanism. In addition, the observed relationships between fertility outcomes and clinical or lifestyle variables are associative rather than causal. This study is presented as a feasibility investigation and the findings should be understood as hypothesis-generating rather than clinically decisive.

Following an extensive literature analysis, details were gathered regarding tactics used for analyzing infertility complications within male healthcare data. The report ends with a summary of the most significant outcomes and conclusions after explaining the methods, dataset properties, and experimental analysis.

## 2 Related studies

The rapid growth of EHRs during the big data era has created significant opportunities for disease risk prediction and clinical decision support. The multivariate time-series data contained in EHRs recording hospital visits, prescriptions, lab tests, and diagnoses of patients offer unprecedented possibilities for clinical state prediction.

### 2.1 EHR-based deep learning for medical event prediction

Modeling temporal dependencies within EHR data remains a primary research focus. Choi et al. (4) proposed Doctor AI, a recurrent neural network (RNN)-based approach that, at each timestamp, receives a multihot vector containing various medical event types and the time interval since the last visit. Baytas et al. (5) presented a T-LSTM, a time-aware LSTM that uses subspace corrosion of LSTM cell retention to deduct memory content based on irregular time intervals. Kwon et al. (6) proposed another RNN-based technique that integrates an attention mechanism called RetainVis. It visits time intervals as additional input features to anticipate the future states of patients. Gao et al. (7) proposed a StageNet model by incorporating time intervals within the LSTM cell and using a convolutional module to capture correlations across neighboring disease progression stages. Jin et al. (8) proposed the HiTANNet model to train global and local temporal information through a hierarchical, transformer-based staged attention approach. However, these techniques do not differentiate between different types of medical events, and they only account for elapsed time.

Liu et al. (9) presented a case study on obesity datasets to demonstrate that the NLP2FHIR representation can be used for clinical phenotyping. Deep learning models improve both clinical data classification and unstructured data standardization. In addition, engaging state-of-the-art deep learning methods, Han et al. (10) methodically built an extensive set using unstructured EHRs to extract Social Determinants of Health (SDOH) standard ontologies. They also showed how DL can automate the removal of important social aspects that impact health results.

In addition, McGilvray et al. (11) created a model to predict when patients with heart failure will die or experience significant decompensation using EHR. To improve patient care, the predictive power of deep learning is used to highlight the significance of commonly available EHR characteristics to aid physicians in making timely decisions. Zhang et al. (12) suggested integrating multimodal longitudinal EHR data to improve the accuracy of predicting multiple sclerosis disease severity. This study demonstrates that combining several forms of data can improve the results. Despite recent improvements, deep learning EHR categorization faces two issues: missing values and data imbalance, which impair the performance of models. Chang et al. (13) obtained trustworthy classification results by controlling noise in EHR datasets through a meta-learning strategy.

Niu et al. (14) used Dirichlet Process Mixture Models and attention mechanism to predict sickness risk based on electronic health records. The non-parametric clustering model maintained good predictive accuracy and increased interpretability. Pedro et al. (15) found that hypertension and hemoglobin are important clinical features for predicting chronic kidney disease using an explainable XGBoost model. However, the model was not tested on a larger population. Martino et al. (16) demonstrated a reasonable model for predicting malnutrition utilizing heterogeneous m-health data and contextualizing m-health-generated risk ratings for the condition, and claimed that longitudinal data are important for customized healthcare.

In addition, XAI tools are models that rely on deep learning. Giovanni et al. (17) used Shapley Additive Explanations (SHAP) to identify glycemic predictors and used a deep neural network to train and forecast postprandial glucose levels in patients with Type 1 Diabetes. However, further testing in a real-world healthcare environment is necessary. Loh et al. (18) conducted a systematic review focusing on the primary functions of SHAP and Grad-CAM to enhance the interpretability of the healthcare model.

### 2.2 Machine learning and quantum machine learning in assisted reproductive technologies and male infertility

Chavez-Badiola et al. (19) developed a model that combines time-lapse morphological annotations with maternal age as predictors within a support vector machine (SVM) classifier for pregnancy outcome classification, achieving an AUROC of 0.75–0.77. Yuan et al. (20) used gradient boosting machine models to non-invasively predict euploidy with an AUC of 0.88, incorporating morphological characteristics such as blastocyst expansion timing, and compared this accuracy against invasive PGT for aneuploidy (PGT-A). Qiu et al. (21) introduced an XGBoost-based live birth prediction model incorporating endometrial thickness alongside other maternal and cycle characteristics rather than relying solely on endometrial thickness as a predictor. The proposed model showed the best calibration when compared to other ML models, with an area under the ROC of 0.73.

Diaz et al. (22) conducted a non-systematic review that provided examples of potential medical applications of ML algorithms in male infertility. These include progressing computer-assisted semen analyses, which examines preoperative images earlier for surgical sperm salvage, intraoperative advancements to detect genetic sensitivity at the microscopic level, and sperm analysis and identification. They also mentioned popular robots and mechanics used for specific analyses. Table 1 summarizes the results of several studies that used AI models to examine MI and the possibility of sperm recovery as a risk element for the condition.

**Table 1:** Summary of the results of several research studies using AI models.

Authors	Utilized algorithms	Utilized data source	Outcomes	Limitations
Ma et al. (23)	Artificial Neural Network (ANN)	Leptin and FSH levels	Predicted NOA sperm recovery from patients with AUC of 0.83	Small, single-center cohort; narrow hormonal predictor set; no external validation
Gil et al. (24)	Decision tree (DT), multilayer perception (MLP), SVM with C4.5 Algorithm	Information on environmental and lifestyle features.	DT attained a specificity of 25%, MLP achieved 69%, and SVM achieved 73.9%	Low specificity for MLP; no explainability; limited feature set
Ramasamy et al. (25)	ANN	Clinical and laboratory data	ANN 59.6% LR:59.4% accuracy:	Low overall accuracy; single-center data; no temporal modeling
Zeadna et al. (26)	Gradient boosting technique (GBT), Multivariate Iogistic Regression Model (MvLRM)	Baseline hormonal sketch (earlier TESE) of serum FSH, T, and LH	Predicted presence of spermatozoa in NOA patients with AUC of 0.807	Retrospective design; hormonal features only; no external cohort
Bachelot et al. (27)	SVM, Random Forest (RF), GBT, extreme gradient boosting (XGB), LR	Testicular sperm extraction with age, tobacco depletion, BMI, FSH, and LH assessment	84.6%: RF 76.9%: GBT and 80.8% XGB performances	No external validation; limited explainability; heterogeneous predictor definitions
GhoshRoy, Alvi, and Santosh (28)	SVM, RF, Naive Bayes, LR, DT, MLP, Sdaboost	Environmental and lifestyle factors	All models achieved an accuracy of more than 80% with the RF classifier, achieving an accuracy of 96.7%.	Small sample; possible overfitting; no temporal/sequential modeling
Li et al. (29)	XGBoost, LightGBM, KNN, naive Bayes, RF, DT	840 patients’ records who underwent ART with 19 features	90.5% accuracy was attained by LightGBM	Single outcome prediction; no multivariate temporal or quantum modeling
Wang et al. (30)	LR, DT, NB, EF, GB, SVM	14,732 clinical data patient’s retrospectives	Attained 0.66, 0.66, and 0.6, of AUCs for predictive performance before treatment reached with pre-eclampsia, placental complications, and postpartum hemorrhage for the first cycle	Modest AUCs; retrospective; no explinability analysis
F Li et al. (31)	LR	281 patients with single-center clinical variables	Attained 0.786 of AUC	Limited sample size from a single cohort; reducing generalizability; limiting its ability to capture complex non-linear relationships among clinical variables
Xignan et al. (32)	LR (multivariate)	1,770 patients with single-center clinical and semen parameters	Attained 0.756–0.776 of AUC	Retrospective single-center study using LR, which may not capture complex non-linear relationships among clinical and semen parameters
Deghan et al. (33)	RF, SVM, ANN, ADB	812 patients Clinical and laboratory variables from a single center	Attained 89.8% of accuracy	Evaluated only conventional ML without DL; no external validation; limited generalizability findings
E Seli et al. (34)	Neural networks	10,774 cycles Clinical predictors	Attained AUC of 0.83–0.86	Large, but single center; limited interpretability; no quantum comparison

### 2.3 Quantum-enhanced and hybrid classical learning models

LaRose et al. (35) presented various quantum-encoding methods, such as angle coding, amplitudes coding, and dense angle encoding. Angle encoding and dense angle encoding require O(N) qubits (linear in the number of classical features). Gao et al. (36) produced comparatively shallow circuits, making them well-suited to near-term (NISQ) hardware, whereas amplitudes encoding requires only O (log N) qubits but at the cost of a more complex quantum state-preparation routine.

Sousa et al. (37) proposed a hybrid quantum-classical convolutional neural network (CNN) with improved feature extraction and adversarial robustness, outperforming classical and CNN on the MIST dataset. Bar et al. (38) proposed a VQC using amplitude encoding to reduce qubit requirements and demonstrated its effectiveness on a navigation problem with improved parameter efficiency and success rate.

Grossi et al. (39) utilized a hybrid quantum traditional model founded on a quantum support vector machine (QSVM) and exhibited higher enactment through the utilization of quantum-enriched feature spaces. Their research demonstrated that quantum kernels might offer enhanced classification accuracy relative to traditional SVMs in complex regions.

Kyriienko et al. (40) also developed an unsupervised quantum ML technique that uses quantum kernels to map traditional characteristics into a larger covert space, which is more expressive than classical radial basis function kernels.

Ahalya et al. (41) used QSVM for Parkinson's disease diagnosis, achieving 85% classification efficacy using QSVM combined with classical ML methods to process large volumes of thermal imaging data.

Chatterjee and Das (42) attained an accuracy of 92.7% for grading breast cancer diagnosis using a Quantum Support Classifier with a novel ensemble clustering model with a voting-based weighted interval fuzzy system.

Jenber Belay et al. (43) achieved an accuracy of 99.89%, with a 99.13% F1-score and 99.99% AUC by combining DL features from ResNet50 and VGG16 models with QSVM for the classification of Alzheimer's based on MRI scans.

However, Bhaskaran and Prasanna (44) used the Canberra distance measure to contrast traditional KNN with QKNN on public datasets, and the results showed that QKNN attained accuracies of up to 93.85% and 93.33%, respectively. Nevertheless, traditional KNN showed a marginal benefit with an accuracy of 96.49%.

Although QML is computationally efficient, Yadav et al. (45) and Chatterjee and Das (42) attained the same level of accuracy as traditional models, which also did not include validation in actual clinical contexts. Therefore, its practical applications are still in the experimental phase. Using a combination of CNNs and SVM, it is computationally expensive and requires optimization before it can be used in practice.

Although these deep learning models demonstrated promising performance in EHR analysis, most studies have primarily focused on general disease prediction rather than reproductive health. Furthermore, many methods have been designed to model temporal relationships alone and have paid limited attention to heterogeneous clinical, lifestyle, and environmental factors that jointly influence male fertility. Several studies also relied on single-center datasets and did not incorporate explainability mechanisms, reducing their clinical interpretability. These limitations motivate the development of a more comprehensive and interpretable framework for predicting male infertility.

### 2.4 Critical analysis and research gap

Overall, the literature indicates three recurring limitations: first, EHR-based deep learning models model temporal event sequences but do not differentiate between event types relevant to fertility outcomes, and none have been applied specifically to male infertility. Second, assisted reproductive technologies (ART) and male infertility-focused ML studies predominantly rely on single-center cohorts, narrow predictor sets (e.g., hormonal panels alone) and lack both external validation and model explainability; none combine comprehensive multivariate lifestyle or clinical EHR data with sequential deep learning. Third, quantum machine learning studies have demonstrated feasibility in other clinical domains but almost universally use the quantum component as a postprocessing feature extractor or kernel, rather than embedding it within a recurrent architecture's internal gating mechanism, and none have been evaluated for fertility-related prediction. The present study addresses all three gaps by applying and benchmarking multiple task relevant LSTM variants on both a multidecade fertility rate series and an EHR-derived multivariate male fertility dataset; proposing a quantum LSTM in which a variational quantum circuit is embedded directly within the LSTM input gate, rather than used as a *post-hoc* feature extraction; and providing SHAP-based explainability and multivariate visual analysis absent from prior male infertility ML studies.

## 3 Methodology

This study addresses the methodological and conceptual gaps in current MI research. It provides a novel integrative perspective for the early diagnosis and risk assessment of male reproductive health. Furthermore, the comparative analysis of classical and Quantum LSTM architectures offers reproducible guidance for future modeling approaches in fertility and other sensitive, small-sample clinical domains. Figure 1 shows the overall workflow of the proposed prediction model.

**Figure 1:**
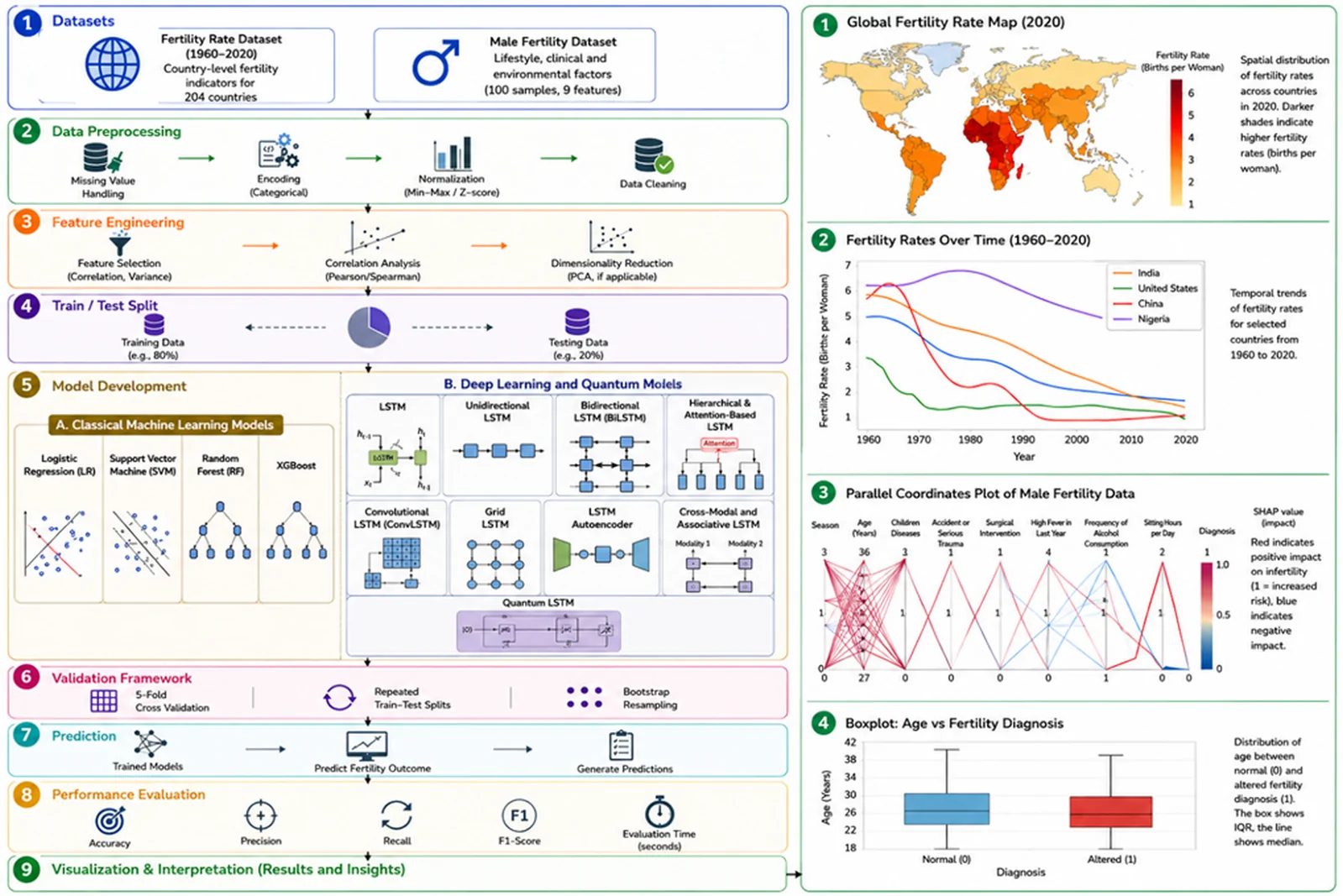
Overall workflow of the proposed prediction model.

### 3.1 Methods

#### 3.1.1 LSTM

LSTM is an RNN technique (4) that aims to model sequential data and overcome the vanishing gradient problem through memory cells and gating methods. LSTM networks have been successfully employed to capture long-term temporal dependencies and are widely used for sequence modeling tasks.

#### 3.1.2 Unilateral LSTM

Similar to traditional LSTM networks, unilateral LSTM processes sequential information in a single temporal direction (46), typically from past to future. Each hidden state depends only on previous observations, making the model suitable for forecasting and real-time sequence prediction (47).

#### 3.1.3 Bidirectional LSTM

The bidirectional LSTM (BiLSTM) (47) consists of two parallel LSTM layers that work in the forward and backward directions of the input sequence. This design is suitable for applications that aim to forecast consequences by relating data from past and future contexts (48). In contrast to unilateral LSTMs, BiLSTM models learn more context by looking at data from both the past and the future.

#### 3.1.4 Hierarchical LSTM and attention-based LSTM

Standard LSTMs may challenge the recognition of interconnections and interdependencies among the stages of complex sequences (49). The use of hierarchical and attention-based LSTMs is a common method for overcoming this restriction.

##### 3.1.4.1 Hierarchical LSTM

Hierarchical LSTM employs multilevels of recurrent processing to learn hierarchical representations from long sequences (50). Lower layers capture local temporal patterns, whereas higher layers model higher-level semantic or contextual information.

##### 3.1.4.2 Attention-based LSTM

An attention-based LSTM integrates an attention machine that allots adaptive weights to different hidden states, allowing the technique to emphasize the most informative time steps when generating predictions (51).

#### 3.1.5 Convolutional LSTM

Convolutional LSTM (ConvLSTM) encompasses the conventional LSTM by changing fully associated procedures with convolutional operations within the gating mechanism (52, 53). This architecture simultaneously models spatial and temporal dependencies and is widely applied to video analysis, weather forecasting, and medical image sequences.

#### 3.1.6 LSTM autoencoder

Autoencoder LSTMs consist of an encoder that constricts an input sequence into a hidden representation and a decoder that rebuilds the inventive sequence (54). It is commonly used for sequence reconstruction, anomaly detection, and unsupervised feature learning (55). A significant reconstruction error (56) indicates non-normality in the input data.

#### 3.1.7 Grid LSTM

Grid LSTM extends the conventional LSTM architecture by introducing recurrent connections across multiple dimensions (57). However, customary LSTMs model temporal dependencies along a single dimension. Simultaneously, this method captures dependencies across multiple spatial and temporal dimensions (58).

#### 3.1.8 Cross-modal and associative LSTM

LSTMs with cross-modal and associative features are specific designs that supplement the normal LSTM to analyze and relate data from many modalities or to execute associative learning tasks (59).

##### 3.1.8.1 Cross-modal LSTM

Cross-modal LSTM is a specialized extension of the conventional LSTM architecture designed for multimodal learning, where information from multiple input modalities is integrated through recurrent processing (60). This method is particularly useful for tasks that require an understanding of relationships among many varieties of data, such as video captioning and emotion recognition. Hence, LSTM setups integrate information from numerous modalities to obtain a combined depiction.

##### 3.1.8.2 Associative LSTM

The associative LSTM is a specialized recurrent architecture that arguments the conventional LSTM (62) memory mechanism with associative memory operations to improve long-range dependency modeling (61).

#### 3.1.9 Various LSTM algorithm workflows

1. Input data preparation
  • Collect your data.
  • Clean and convert it into sequences.
  • Normalize or scale the values if needed.
2. Model selection
  • Choose the appropriate LSTM type:
    ○ Unilateral LSTM: if only past data are required.
    ○ Bidirectional LSTM: if both past and future contexts are required.
    ○ Hierarchical LSTM: for long and complex sequences.
    ○ Attention-based LSTM: to emphasize significant quantities of input.
    ○ ConvLSTM: for images or video sequences.
    ○ Autoencoder LSTM: for anomaly detection or sequence reconstruction.
    ○ Grid LSTM: for multidimensional sequences.
    ○ Cross-modal/associative LSTM: for combining multiple data types.
3. Model building
  • Create an LSTM model using layers.
  • Add extra layers for attention and convolution algorithms.
  • Set parameters such as the number of batch size, units, and learning rate.
4. Training the model
  • Feed the sequence data into the model.
  • The model acquires patterns from information step-by-step.
  • It adjusts itself by comparing the predicted results to the actual ones.
5. Making predictions
  • Once trained, use the model to predict future values or detect patterns.
  • Use the model to forecast, classify, or detect anomalies.
6. Evaluating the results
  • Measure how well the model performs using metrics including F1-Score, Accuracy, Precision, Recall, and Time.

#### 3.1.10 Quantum LSTM

##### 3.1.10.1 Classic quantum computing

Quantum computing utilizes concepts from quantum mechanics, such as entanglement and superposition, to perform calculations on quantum hardware that operates using qubits rather than classical bits. These quantum computers process enormous quantities of information simultaneously, allowing them to solve complex problems much faster than traditional computers for exact tasks such as optimization, search, and quantum simulation. To understand how LSTM can be used in a quantum setting, several fundamental concepts of quantum computing, such as Hadamard gates and qubits, must be understood.

- A qubit is a superposition of states signifying values |0⟩ or |1⟩ or any quantum superposition thereof. As defined in Equations 1, 2,
  |φ⟩=α|0⟩+β|1⟩ (1)

α,βϵC denotes the complex value amplitudes of the quantum state.

$$|\alpha {|}^{2}+|\beta {|}^{2}=1\;\text{signifies conditional normalization}$$ (2)

- Hadamard gate: A superposition creation defined as
  $$H=\;\frac{1}{\sqrt{2}}\left[\begin{array}{cc} 1 & 1\\ 1 & -1 \end{array}\right]$$ (3)

After applying the Hadamard gate, the qubit has a *50% chance* of being measured as 0 or 1. This operation is a common first step in several quantum procedures.

- Variational quantum circuit: A type of quantum circuit utilized mainly for *learning and optimization tasks* that combine quantum and classical computing. The structure is as follows:
  1. Initial state preparation: This often starts with qubits in the ∣0⟩ state and then Hadamard gates are applied to create a superposition.
  2. Parameterized quantum gates: These gates have tunable parameters (angles), such as R_x(*θ*), R_y(*θ*), or R_z(*θ*). A classical computer optimizes these parameters.
  3. Measurement: The qubits are measured to obtain results (expectation values).
  4. Classical optimizer loop: A classical algorithm such as LSTM or Adam is used to update the parameters and improve performance.

##### 3.1.10.2 Quantum LSTM

The quantum LSTM model has several parts that work together to process sequential inputs efficiently and effectively. The Quantum Embedding Layer starts the process by converting classical input information into quantum states. The Quantum Memory Cell subsequently receives the encoded data. Quantum gates retain information and updates the states. The classical-quantum interface is crucial for enabling communication between the classical and quantum parts. It encodes standard information into quantum positions and decodes quantum states back into classical data. Each part has a specific job to perform and is designed to operate well with the others. This method uses the characteristics of quantum computing to improve feature representation learning more efficiently.

##### 3.1.10.3 Architecture of Quantum LSTM

- Input: sequence X with shape (batch, T, n_features_reduced).
- Classical embedding: a modest thick layer that maps features to a dimension that matches the sum of qubits or the input circuit.
- Quantum LSTM cell: For every time step t:
  ○ Encode x_t into a parameterized quantum circuit using angle encoding.
  ○ Use a VQC with learnable parameters, such as variation rotations and entangling gates.
  ○ Measure the expectancy values of selected observables, such as Pauli Z, for each qubit to generate a tiny classical vector q_t.
  ○ Combine q_t with the preceding hidden state (classically) using LSTM-like gating that is performed in classical layers but uses q_t instead of classical linear transforms. Alternatively, use quantum gates to generate gating signals (more sophisticated).
  ○ Output the classical tensors h_t and c_t for unknown and cell states.
- Stacking: stacks more than one QLSTM layer or switch between classical LSTM and QLSTM layers.
- Head: a traditional feedforward network that maps the final hidden state to the target for classification purposes.

Two common methods for creating Quantum LSTM are as follows:

- Quantum feature extractor inside LSTM: employ a VQC instead of the LSTM input-to-hidden linear transform to output features that classical gates use.
- Quantum LSTM cell: use VQC outputs to determine how to activate gates, which remain classical.

#### 3.1.11 Friedman statistical test

The Friedman non-parametric test was used to compare multiple LSTM architectures across the repeated validation runs. The test evaluates whether the observed performance differences are statistically significant without assuming the normality of the data, as defined in the following equation:

$$\gamma_{F}^{2}=\frac{12N}{k(k+1)}\left(\overset{k}{\underset{\;j=1}{\sum }}\;R_{j}^{2}-\frac{k{\left(k+1\right)}^{2}}{4}\right).$$ (4)

where *N* is the number of validation runs, *k* is the number of classifiers, and $R_{j}$ is the average rank of the *j*th classifier.

#### 3.1.12 Wilcoxon signed rank test

The Wilcoxon signed-rank test is a non-parametric statistical test for comparing two related models when the data are not always normally distributed. The Wilcoxon signed-rank test statistic is computed, as in the following equation:

$$W=\min(W^{+},\;W^{-})$$ (5)

where $W^{+}$ is the positive differences in sum ranks, and $W^{-}$ is the negative differences in sum ranks.

The corresponding *P*-value is utilized to regulate the statistical implication. A *P*-value <0.05 indicated a statistically significant difference between the two models.

The effect size was computed as in the following equation:

$$\tau =\;\frac{\left|Z\right|}{\sqrt{n}}$$ (6)

where *Z* is the standardized Wilcoxon statistic, and *n* is the number of paired observations.

Table 2 summarizes the advantages, limitations, and key architectural parameters of classical LSTM variants.

**Table 2:** Comparative summary of classical LSTM variants.

Model	Key architectural parameters	Advantage	Limitation	Typical application
LSTM	Single lSTM layer (64 hidden units), tanh activation, sigmoid gates, dropout = 0.2	Learns long-term temporal dependencies and mitigates the vanishing gradient problem	Captures data only in the forward direction	General sequence modeling
Unilateral LSTM	One forward LSTM layer (64 units), dropout = 0.2	Computationally efficient with fewer parameters	Cannot utilize future temporal information	Time-series forecasting, anomaly detection
Bidirectional LSTM	Forward and backward LSTM layers (64 units each), concatenated outputs	Exploits both past and future contextual information	Higher computational cost and requires complete sequences	Sequence labeling, contextual prediction
Hierarchical and Attention-based LSTM	Two-level stacked LSTM architecture (64 units per layer); LSTM (64 units) with soft-attention layer	Learns hierarchical temporal representations; automatically concentrates on the most informative time steps	Increased architectural complexity and training time; additional trainable parameters increase thecomputational cost	Long, structured sequences; long sequences with salient subevents
Convolutional LSTM	1D convolution layer (kernel size = 3) followed by LSTM (64 units)	Learns local feature interactions before temporal modeling	Primarily designed for spatial or spatiotemporal data; limited benefit for tabular datasets	Spatiotemporal data (images/video); tested here as baseline
LSTM Auto Encoder	Encoder (64 units) + decoder (64 units)	Learns compact latent features, represents, and reconstructs sequences	Reconstruction objective is not directly optimized for classification	Anomaly detection, sequence reconstruction
Grid LSTM	2D grid, 64 units/cell	Models multidimensional dependencies	Higher complexity; less established	Multidimensional/structured data
Cross-Modal and Associative LSTM	2 parallel 64 unit encoders + fusion/key-value memory	Integrates multiple modalities or associations	Requires modality-aligned data	Multimodal fusion, associative recall tasks
Quantum LSTM	64 unit LSTM; VQC (8 qubits, 2 variational layers) embedded in the input gate	Quantum superposition/entanglement enrich gate representations; lower variance on small data	Simulator-based; modest accuracy gain; added implementation complexity	Small, heterogeneous clinical datasets (feasibility study)

### 3.2 Datasets

#### 3.2.1 Dataset 1: Fertility rate

Fertility rate data from 1960 to 2020 were sourced from the Kaggle website (https://www.kaggle.com/datasets/elmoallistair/fertility-rate-19602020/data). In any given year, the total fertility rate is the sum of all births that would occur if all women lived to their reproductive age and followed the current age-specific fertility rates. This rate comes from the population and should remain relatively steady with a total fertility rate of 2.1 children per woman, presuming no net migration and unchanged mortality. Fertility, a component of population development alongside migration and mortality, reflects the causes and effects of social and economic changes. The postponement of family formation, the delay in childbearing, and a reduction in preferred family size have collectively contributed to a significant decline in birth rates over the past decades. The birth rate was used as a proxy for this indicator. The dataset parameters are depicted in Table 3.

**Table 3:** Characteristics of fertility rate dataset.

S.No.	Parameter	Description	Data type	Unit
1.	Country	Country/region	Categorical	—
2.	1960–2020	Annual fertility rate	Continuous	Births per woman

#### 3.2.2 Dataset 2: Fertility of men

This dataset was obtained from the Kaggle website (https://www.kaggle.com/code/tanshihjen/factors-influencing-semen-concentration/input); however, it was primarily obtained from the UCI machine learning repository. The dataset consisted of 100 participants who had their sperm examined in accordance with the World Health Organization's 2010 standards. Using 100 samples and 10 features that indicate sperm normality or abnormality, the UC Irvine machine learning repository correlates sperm concentrations with demographic and social data, environmental factors, health status, and lifestyle choices. The features that were recognized included age, season, childhood diseases, daily sitting duration, diagnosis, regularity of alcohol intake, high fevers in recent years, surgical intervention, accidents or major traumas, and surgical procedures. The dataset parameter description is shown in Table 4.

**Table 4:** Characteristics of male fertility.

S. no.	Parameter	Description	Data type	Unit	Minimum	Maximum
1.	Season	Season of assessment	Categorical	—	Spring	Winter
2.	Age	Patient age	Integer	Years	27	36
3.	Childhood diseases	Childhood disease history	Binary	Yes/no	No	Yes
4.	Accident or serious trauma	History of trauma	Binary	Yes/no	No	Yes
5.	Surgical intervention	Previous surgery	Binary	Yes/no	No	Yes
6.	High fevers in the last year	Fever history	Ordinal	Category	None	High
7.	Frequency of alcohol consumption	Alcohol intake frequency	Ordinal	Category	Never	Frequent
8.	Smoking habit	Smoking status	Binary	Yes/no	No	Yes
9.	Number of hours spent sitting per day	Daily sedentary time	Integer	Hours/day	1	342
10.	Diagnosis	Fertility status	Binary	Class label	Normal	Altered

### 3.3 Data preprocessing

The aim of the data groundwork was to create a dataset ready for further analysis. Both datasets underwent independent cleaning and preprocessing steps.

#### 3.3.1 Fertility rate dataset

Initially, missing years were removed from countries with incomplete temporal sequences. Normalized fertility values were obtained using Min–Max scaling (0–1). Time-indexed sequences were converted into fixed-length sliding windows (length = 10 years) to model temporal patterns.

#### 3.3 2 Male fertility dataset

In this dataset, categorical variables were encoded using one-hot encoding (season, alcohol frequency, and smoking habit) and label encoding for binary variables. Numerical features (age and sitting hours) were scaled using a Standard Scaler. Multicollinearity was checked using Pearson’s correlation (<0.85 retained). Class imbalance was handled using random undersampling to preserve model generalizability.

## 4 Experimental analysis

Data were gathered in the initial phase and processed through a preprocessing flow that included extensive cleaning of two separate datasets: the fertility rate and fertility of men datasets individually. Next, the essential library files were installed in Python to train the two datasets. In this context, the quantity and size of the two datasets are limited because the chosen topics of human fertility and infertility is very sensitive and restricted. Therefore, our research leverages the available data to address this gap. Although the dataset size is limited, its small scale facilitates data inspection and quality verification. These two datasets were trained using basic machine learning and various LSTM models to provide a fair comparison. This is because of the unique characteristics of buildings, temporal connections, and specific environments for prediction, as well as the unusual nature of the analytical tasks. These objectives can be accomplished by first splitting the datasets into training and testing sets. For our analysis, the dataset was split into training and testing sets as 80% and 20%, respectively. The overall experimental setup is shown in Tables 5a,b.

**Table 5a:** Experimental setup and reproducibility details of the LSTM.

Parameter	Description
Programming Language	Python
Deep Learning Framework	TensorFlow and Keras
Environment	Google Colab
Random Seed	42
Validation Strategy	Fivefold Cross Validation
Repeated Train-Test Splits	80–20
Bootstrap Resampling	1,000 Iterations
Entanglement Strategy	Strongly Entangling Layers
LSTM Hidden Units	64
Batch Size	32
Epochs	20
Optimizer	Adam
Learning Rate	0.001
Loss Function	Binary Cross-Entropy, Mean-Squared Error
Early Stopping	Patience = 10 Epochs
Hyperparameter Tuning Method	Grid Search With Cross-validation
Evaluation Metrics	Accuracy, Precision, Recall, F1 Score, Training Time

**Table 5b:** Experimental setup and reproducibility details of the quantum-inspired model.

Parameter	Description
Quantum Computing Model	PennyLane
Quantum Gates	RX,RY,RZ,CNOT
Quantum Simulator	PennyLane Lightning Qubit
Number of Qubits	8
Quantum Circuit Depth	3 Variational Layers
Quantum Trainable Parameters	64
Quantum Embedding Method	Angle Embedding
Quantum Layer Placement	Between LSTM Layer and Output Layer
Quantum Measurement	Pauli-Z Expectation Values
Evaluation Metrics	Accuracy, Precision, Recall, F1 Score, Training Time
Explainability Method	SHAP (Shapley Additive exPlanations)
Statistical Validation	Friedman Test, Wilcoxon Signed-rank test, Confidence Intervals, Standard Deviation

### 4.1 Applying basic machine learning algorithm

To establish a robust baseline performance and assess the suitability of deep learning architectures, four conventional machine learning algorithms, namely Logistic Regression, XGBoost, Random Forest (RF), and SVM, were applied to both the fertility rate dataset and the fertility of men dataset. These algorithms were selected because they are widely recognized as effective approaches for structured and tabular data, particularly in small-sample medical prediction tasks. First, LR was employed as a linear probabilistic classifier to provide an interpretable baseline. RF was utilized for its ability to capture non-linear relationships through ensemble decision trees, while mitigating overfitting. SVM was designed for its efficiency in complex classification and to classify optimal decision limits. XGBoost was included because of its strong predictive capability and robustness in handling heterogeneous clinical and demographic data. The model performance was assessed using precision, accuracy, recall, F1 score, and computational time. The performance of the model is shown in Tables 6a,b.

**Table 6a:** Training fertility rate dataset using a basic machine learning model.

S. no.	Algorithms	Fertility rate dataset			
		Precision (%)	Recall (%)	Accuracy (%)	F1 score (%)
1.	Logistic regression	85.2	84.8	87.0	84.7
2.	Random forest	87.4	86.3	88.3	86.5
3.	XGBoost	87.6	86.9	89.4	87.2
4.	SVM	85.3	84.6	86.1	85.3

**Table 6b:** Training fertility of the men dataset using a basic machine learning model.

S. No.	Algorithms	Fertility of men			
		Precision (%)	Recall (%)	Accuracy (%)	F1 score (%)
1.	Logistic egression	86.2	85.8	88.0	85.9
2.	Random Forest	88.1	87.6	89.2	87.8
3.	XGBoost	88.5	88.3	89.7	88.6
4.	SVM	86.5	85.9	87.8	86.1

As shown in Tables 6a,b, of all the standard machine learning models tested on the fertility rate dataset, XGBoost outperformed them all with an impressive accuracy of 89.4%, recall of 86.9%, precision of 87.6%, and F1 score of 87.2%. Although SVM and LR showed relatively poor prediction performance, RF achieved 88.3%, placing it second. Similarly, for the male fertility dataset, XGBoost again achieved the strongest performance among the baseline models with an accuracy of 89.7%, precision of 88.5%, recall of 88.3%, and F1 score of 88.6%. RF also demonstrated competitive results, with an accuracy of 89.2%. These findings confirm that ensemble-based machine learning methods remain strong competitors for small datasets. In contrast, LSTM-based architectures are capable of learning sequential fertility patterns across multiple decades, thereby providing additional insights into long-term demographic dynamics.

### 4.2 Applying various LSTM and quantum LSTM

To assess the suitability of various DL architectures were implemented on both datasets. Different LSTM models were used because our fertility rate dataset has one type of data, while the men's fertility dataset has multiple types, and they are small datasets. Various LSTM models such as LSTM, Unilateral LSTM, Bidirectional LSTM, Convolutional LSTM, Hierarchical and Attention-based LSTM, LSTM Auto encoder, Grid LSTM, and Cross-modal and Associative LSTM, and Quantum LSTM were applied individually to two datasets with hidden units of 64, activation with tanh (LSTM), ReLU (dense layers), dropout at 0.2, Adam optimizer, a 0.001 learning rate with a 32 batch size, epochs 20 with early stopping (patience = 7) and stated in Python for analyzing complex, heterogeneous, and sensitive data.

However, various LSTMs have different workings on the dataset. Therefore, we first applied basic LSTM, which uses gates such as input, forget, and output to prevent gradients from vanishing. The Unilateral LSTM captures past dependencies in sequences, and the bidirectional LSTM captures relative data from both past and future positions in the input data. Hierarchical and attention-based LSTM processes multiple hierarchical levels and focus on relevant parts because this is rational for the dataset, as EHR features can be grouped into physiological versus behavioral features, making this algorithm's application meaningful. Although convolutional LSTM was traditionally applied to spatial and temporal data, it was included in this study as an exploratory baseline to evaluate whether localized feature interactions could improve fertility prediction. The model was selected not because the datasets contained image information, but to provide a comparative assessment of different recurrent architectures under identical experimental conditions. The LSTM autoencoder reduces the data size and reconstructs sequences, and grid LSTM handles data with multiple dimensions, including architectural stress tests for 2D feature representation of EHR records. Cross-Modal and Associative LSTM use multiple data modalities and retrieve key-value pairs.

Quantum LSTM was executed on the Python platform using the simulator PennyLane to build an LSTM model along with a quantum layer with 8 qubits encodings: angle encoding (Rx/Ry rotations) for input embedding. Variational layers: two layers with CNOT entanglement gates. Circuit depth:18 gates per forward pass. Total tunable quantum parameters: 64 (eight rotational angles * two layers * four qubits per layer). Quantum layer placement: the quantum circuit replaced the input to the hidden weight matrix in the LSTM input gate only. All other LSTM gates (forget, output, and cell) remained classical. This is the key architectural distinction from prior hybrid models that use quantum circuits only as postprocessing feature extractors. The PyTorch and TensorFlow libraries were used because quantum circuits replace some neural layers inside the LSTM. The optimizer is Adam with a batch size of 32, and the learning rate is 0.001 with 20 epochs according to the architecture of Quantum LSTM.

Both datasets were trained with only 20 epochs because of the small dataset size. To ensure that the proposed LSTM and Quantum LSTM models were both accurate and generalizable, three validation strategies, such as fivefold cross-validation to reduce bias, repeated random training-test splits for measuring variance and stability and bootstrap resampling for quantifying uncertainty with confidence intervals (CIs), were implemented.

#### 4.2.1 Cross-validation with k-fold (k = 5)

This cross-validation with a fivefold procedure was applied to both datasets, and the split-trained part dataset was validated on the remaining data repeatedly across five mutually exclusive folds. This allowed the performance of each LSTM architecture, including Unilateral LSTM, Hierarchical and Attention-LSTM, Bidirectional LSTM, Conv-LSTM, Grid-LSTM, LSTM Autoencoder, Cross-Modal LSTM, and the proposed Quantum LSTM, to be evaluated under different data partitions. Across folds, Quantum LSTM consistently achieved the highest accuracy**,** followed by the Unilateral and Bidirectional LSTM models.

#### 4.2.2 Repeated random train–test splits (*n* = 10)

To further reduce the dependence on split data, 10 repeated train–test evaluations were performed, each using a fresh 80/20 split. This method verified the consistency of the model's predictions while capturing the inherent unpredictability due to an imbalanced sample. Compared with classical LSTMs, the Quantum LSTM maintained a lower variance in accuracy, recall, precision, and F1score, suggesting that its hybrid quantum-inspired representation was better able to deal with data noise and heterogeneity. There was low variability in models such as Grid-LSTM and Conv-LSTM; however, LSTM autoencoders were more unstable because they relied on reconstruction-driven learning, which is not ideal for classification jobs.

#### 4.2.3 Bootstrap resampling (1,000 iterations)

Bootstrap resampling was implemented to compute CIs for the performance metrics. In each bootstrap iteration, the prototype was trained on a resampled subsection of the dataset, and predictions were evaluated on out-of-bag samples. This allowed the estimation of a 90% CI for the classification accuracy. The Quantum LSTM demonstrated the narrowest confidence intervals, confirming its strong reliability, even with small sample sizes. Traditional LSTM variants displayed wider CIs, reflecting their greater sensitivity to sample composition.

Although this study used fivefold cross-validation, repeated random train–test splits, and bootstrap resampling for internal validation, the final performance reporting focused on accuracy, recall, precision, and F1 score because these metrics directly quantify how the model classifies the outcome. Therefore, here, we prioritized accuracy, recall, F1 score, and precision. The evaluation of several LSTM models applied to both datasets is noted independently in Tables 7a,b.

**Table 7a:** Training fertility rate dataset with several LSTM models.

S. No.	Algorithms	Fertility rate dataset				
		Precision (%)	Recall (%)	Accuracy (%)	F1 score (%)	Time (s)
1.	LSTM	88.1	88.3	89.4	88.3	91
2.	Unilateral LSTM	90.3	89.8	91.2	89.8	79
3.	Bidirectional LSTM	89.6	89.4	90.2	89.6	80
4.	Hierarchical and attention-based LSTM	89.5	89.7	90.4	89.5	83
5.	Convolutional LSTM	89.2	89.1	90.1	89.1	87
6.	LSTM autoencoder	87.1	87.4	89.6	87.3	89
7.	Grid LSTM	86.3	88.2	89.2	88.2	82
8.	Cross-Modal and associative LSTM	87.5	88.1	89.8	87.5	80
9.	Quantum LSTM	90.6	90.9	92.6	90.7	74

**Table 7b:** Training fertility of the men dataset with several LSTM models.

S. no.	Algorithms	Fertility of men				
		Precision (%)	Recall (%)	Accuracy (%)	F1 score (%)	Time (sec)
1.	LSTM	87.3	87.5	90.2	87.3	93
2.	Unilateral LSTM	90.3	89.6	92.1	90.3	82
3.	Bidirectional LSTM	89.5	88.9	90.6	88.6	85
4.	Hierarchical and Attention-based LSTM	89.2	86.3	90.3	88.1	87
5.	Convolutional LSTM	89.6	89.2	90.7	89.5	89
6.	LSTM autoencoder	88.1	87.1	89.8	89.2	90
7.	Grid LSTM	88.2	89.5	90.3	88.2	92
8.	Cross-Modal and associative LSTM	86.3	87.1	89.6	89.2	86
9.	Quantum LSTM	91.7	90.3	93.8	91.4	79

As shown in Tables 7a,b and Figure 2, from several LSTM models, the unilateral LSTM is the best-performing model for training the fertility rate and fertility of men datasets individually. The fertility rate dataset achieved 91.2% accuracy with a run time of 79  s, whereas for the fertility of men dataset, 92.1% accuracy with a run time of 82 s was achieved, compared to other applied LSTM models, as per our observation. The proposed Quantum LSTM achieved the highest performance compared to various LSTM models, with an accuracy of 92.6% and a lower computational time of 74 s for the fertility rate dataset. For the fertility of men dataset, an accuracy of 93.8% and a lower computational time of 79 s were obtained.

**Figure 2:**
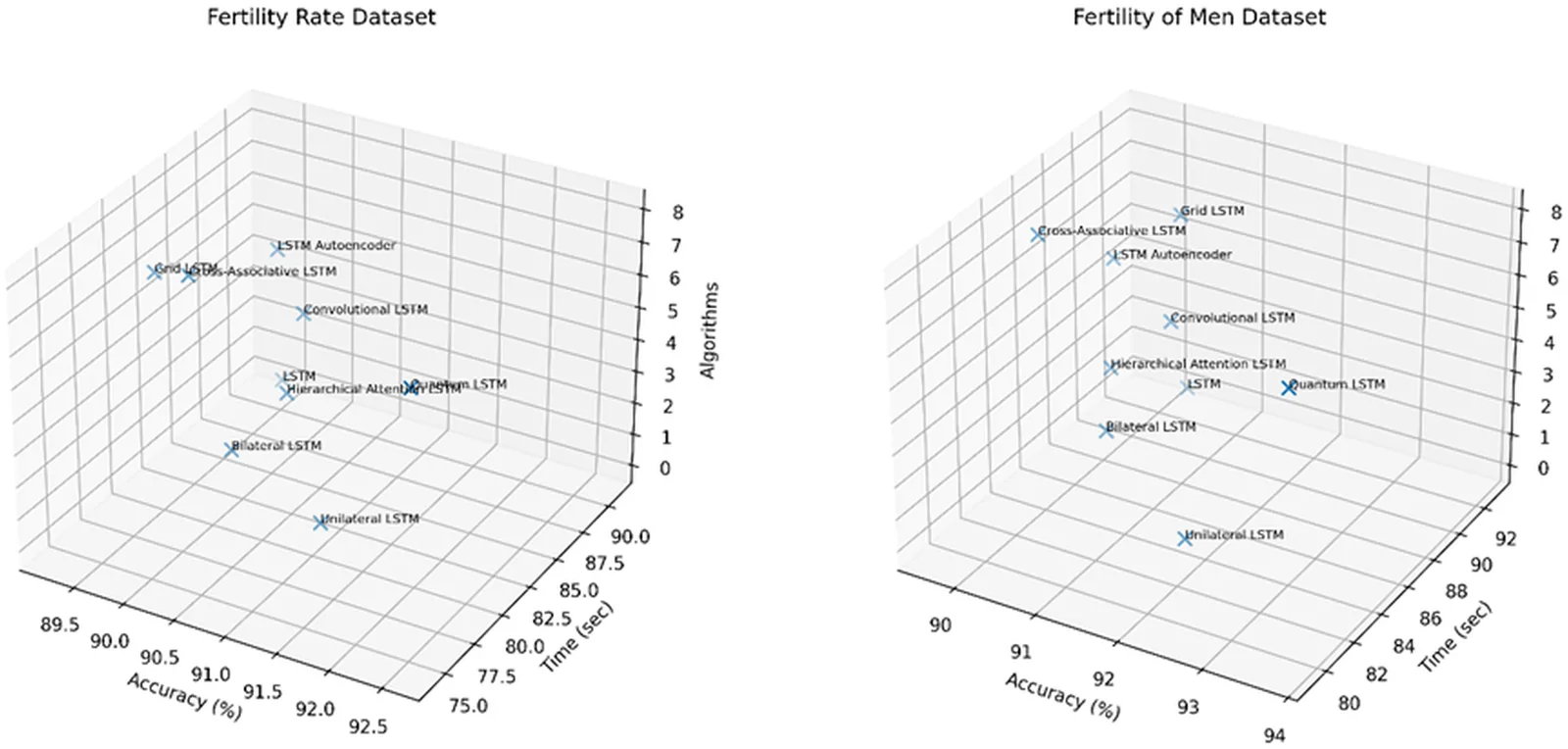
Visualization of various LSTM model trainings.

By observing the accuracy of all applied algorithms for the two datasets, the Unilateral LSTM and Quantum LSTM achieved an accuracy with a variance of 1%–2%. Therefore, to determine the model robustness and reliability, the standard deviation and confidence interval were computed. The confidence interval and standard deviations are as shown in Tables 8a,b.

**Table 8a:** CI and SD for the unilateral and quantum LSTM for the fertility rate dataset.

S. no.	Model	Accuracy (%)	Mean ± SD	90% CI
1.	Unilateral LSTM	91.2	91.2 ± 1.08	90.1–92.3
2.	Quantum LSTM	92.6	92.6 ± 0.84	91.4–93.2

**Table 8b:** CI and SD for the unilateral and quantum LSTM for the fertility of the men dataset.

S. no.	Model	Accuracy (%)	Mean ± SD	90% CI
1.	Unilateral LSTM	92.1	92.1 ± 1.45	90.2–93.8
2.	Quantum LSTM	93.8	93.8 ± 0.92	92.5–94.9

Tables 8a,b present Quantum LSTM achieving the highest average accuracy of 92.6% with a lower SD (± 0.84) compared to unilateral LSTM (± 1.08) for the fertility rate dataset. However, for the fertility of men dataset, an accuracy of 93.8% was achieved with a lower SD (± 0.92) compared to the unilateral LSTM (± 1.45), indicating a more stable performance across different data partitions. To determine whether the experimental performance variances among the assessed LSTM architectures were statistically significant, the Friedman non-parametric test was performed on both datasets to compare multiple LSTM architectures across repeated validation experiments. The test revealed significant variance among the models (*P* < 0.05). The statistical values for both datasets are shown in Table 9.

**Table 9:** Friedman test results for both datasets.

S. no.	Dataset	Test	Statistic	*P*-value
1.	Fertility rate	Friedman	22.42	0.0009
2.	Fertility of men	Friedman	24.81	0.0012

As shown in Table 6, the performance of the evaluated LSTM architectures for the fertility rate dataset yielded a statistical value of 22.42 with a *P*-value of 0.0009. However, for the fertility of men dataset, the test yielded a statistical value of 24.81 with a *P*-value of 0.0012. As both *P*-values are less than 0.05, the null hypothesis of equal classifier performance is rejected. Then, the Wilcoxon signed-rank test was performed to compare pairwise between the unilateral LSTM and proposed Quantum LSTM. Later, the effect size was observed to differentiate the paired observations with interpretation. The baseline comparison of the Wilcoxon signed-rank test and effect size calculation of the best and proposed algorithms for both datasets are shown in Table 10.

**Table 10:** Wilcoxon signed-rank test and effect size comparison for both datasets.

S. no.	Dataset	Test	*P*-value	Effect size (Cohen’s *d*)
1.	Fertility rate	Wilcoxon signed-rank test (fivefold CV)	0.031	0.42 (small to moderate)
2.	Fertility of men	Wilcoxon signed-rank test (fivefold CV)	0.024	0.48 (small to moderate)

As shown in Table 10, pairwise Wilcoxon signed-rank tests compare quantum LSTM with unilateral LSTM across fivefold cross-validation results and demonstrate statistical significance on both datasets (*P* < 0.05). The effect sizes were small to moderate (Cohen's *d* of 0.42 and 0.48), consistent with the reported modest absolute accuracy gain of1%–2%. These results indicate statistically significant differences among the compared models, and the proposed Quantum LSTM exhibited superior performance. Therefore, the observed improvements are statistically supported, although they should be interpreted cautiously because of the limited dataset size.

### 4.3 Explainability analysis

To enhance model interpretability, SHAP analysis was conducted on the both datasets, such as the fertility rate and fertility of men dataset to measure the contribution of each input parameter to the model predictions. SHAP assigns an importance value to every feature by estimating its marginal involvement in the prediction based on cooperative game theory. An explainability analysis was performed on the final trained prediction model using the SHAP Python library. A representative subset of the training dataset was used as the background dataset for estimating the expected model output. The feature importance was calculated using the mean absolute SHAP values across all test samples. The features were ranked in descending order according to their mean absolute SHAP values, where higher values indicated a superior contribution to the prediction.

#### 4.3.1 Fertility rate dataset

The fertility rate time series was transformed into supervised learning sequences, and SHAP values were computed to quantify the involvement of individual temporal features in model predictions. The prediction of important features is shown in Figure 3.

**Figure 3:**
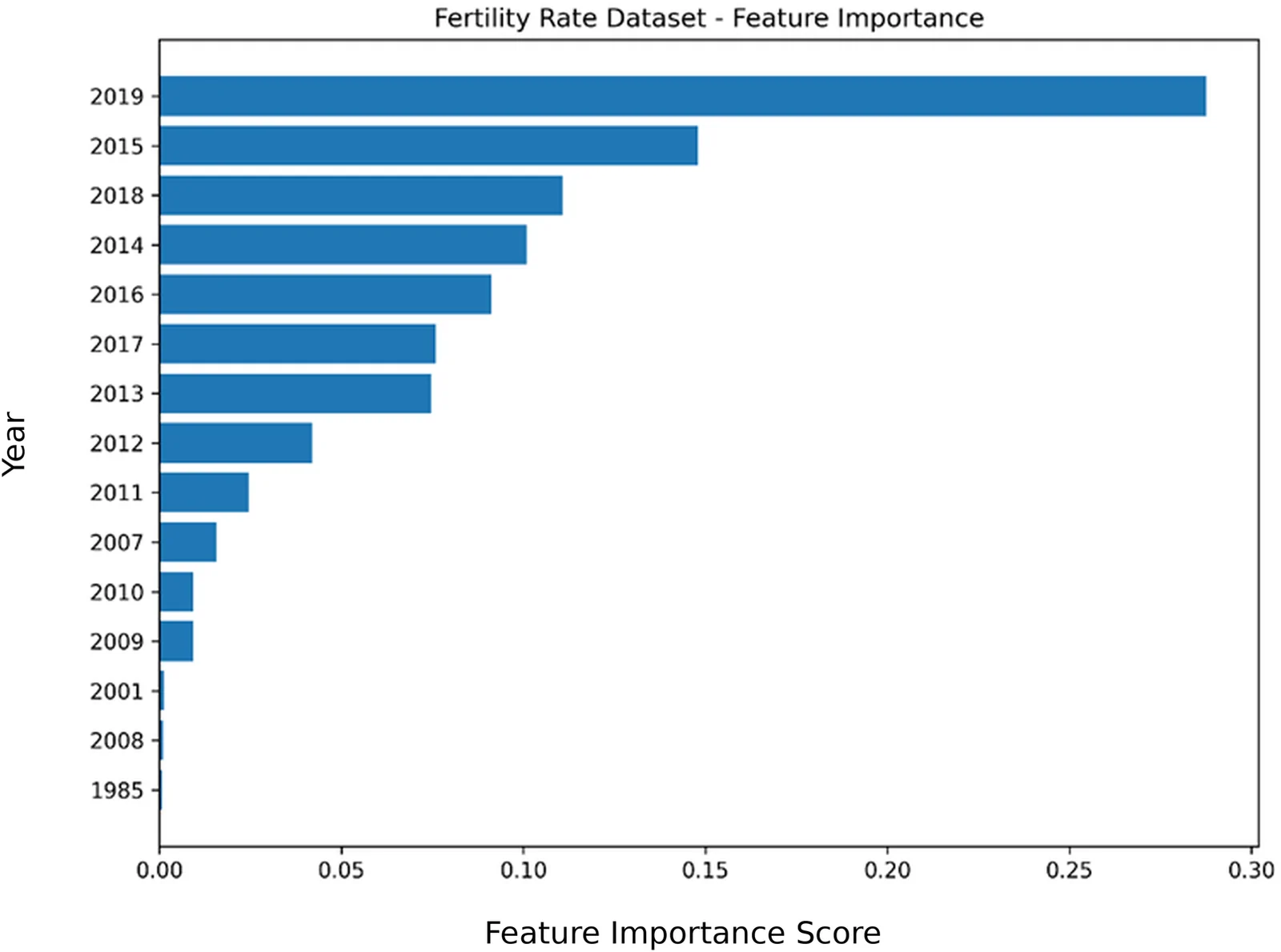
Feature importance analysis of fertility rate.

As shown in Figure 3, the 2019 fertility rate has the highest feature importance of approximately 0.29, indicating that it contributes the most to the model's predictions. The fertility rates for 2015, 2018, 2014, and 2016 also exhibited relatively high importance values, suggesting that these years provided substantial predictive information. In contrast, earlier years such as 2001, 2008, and 1985 had very low SHAP values, indicating a comparatively smaller contribution to the model's overall predictions.

#### 4.3.2 Fertility of the male dataset

The analysis recognized the most influential variables contributing to fertility prediction and provided insight into the decision-making behavior of the predictive model's variables with higher importance scores, which contributed more strongly to classification outcomes, indicating their greater predictive relevance within the dataset, as shown in Figure 4.

**Figure 4:**
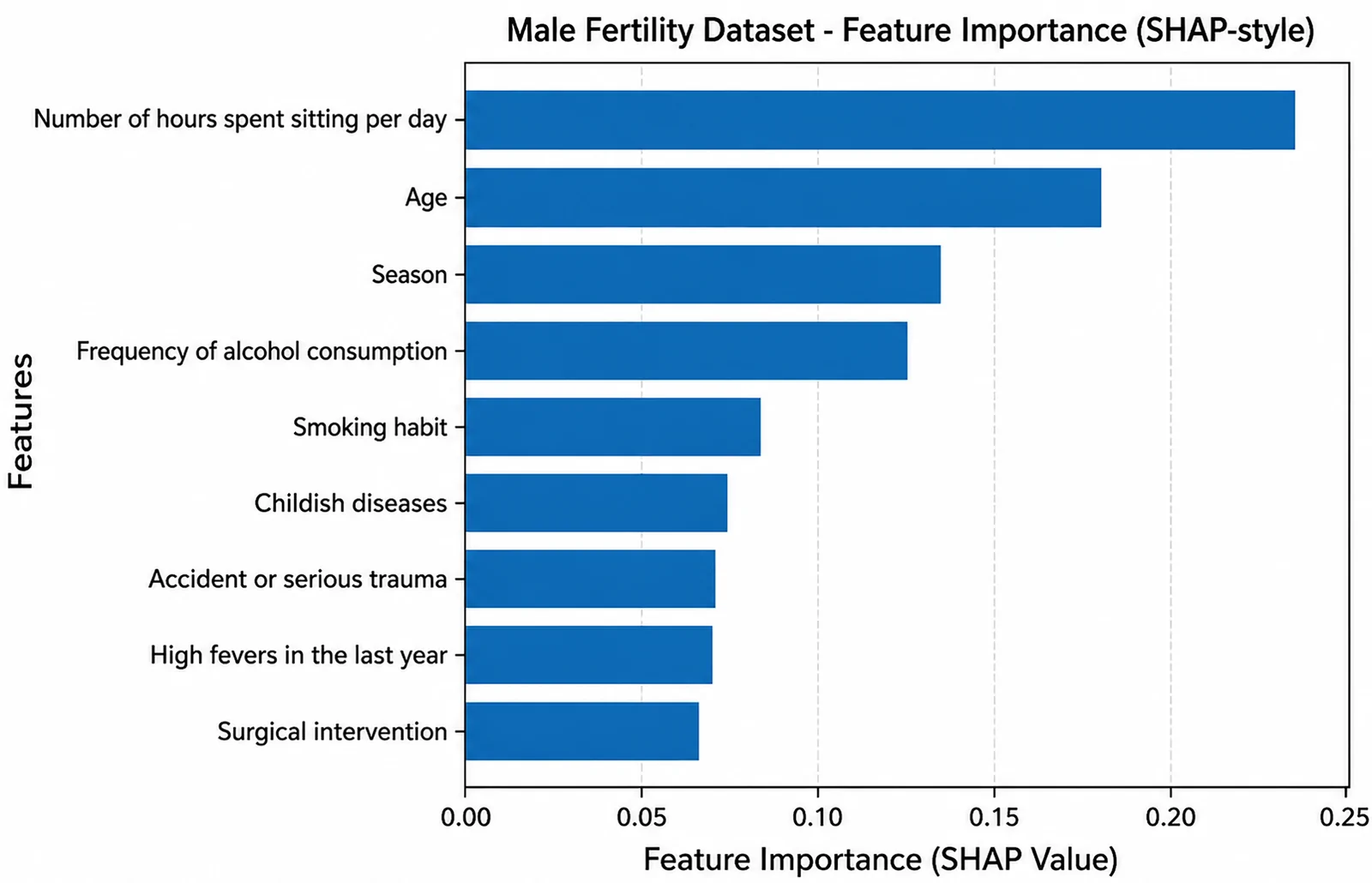
Feature importance analysis of men fertility.

Figure 4 shows the ranked features according to their mean absolute SHAP values, where higher values indicate a superior influence on the prediction model. Among all variables, the number of hours spent sitting per day exhibits the highest importance score (approximately 0.22), indicating that sedentary behavior has the greatest influence on the model predictions. Age is the second most influential feature (approximately 0.18), suggesting that age-related physiological changes play a significant role in fertility prediction. The next most important variables are the season and frequency of alcohol consumption with importance scores of approximately 0.13 and 0.12, respectively. These findings indicate that environmental and lifestyle-related factors contribute substantially to the predictive performance of the model.

Clinical history variables, including smoking habits, childhood diseases, accidents or serious trauma, high fevers in the last year, and surgical intervention, exhibit comparatively lower importance values, ranging from approximately 0.07 to 0.08. Although these variables contribute less individually, they still provide complementary information that improves the overall predictive capability of the model.

Therefore, the gradual decline in SHAP importance across older observations suggests that the model relies on both short- and long-term temporal dependencies while generating predictions.

After the dataset was trained with several LSTMs and the proposed quantum LSTM model and interpreted with the SHAP model, the following predictions were made at each stage of the analysis after applying the technique separately.

### 4.4 Dataset 1: Fertility rate

First, a global overview of fertility rates by country is visualized because it helps policymakers, researchers, and public health experts understand trends, make decisions, and plan interventions at the national and international levels. Figure 5 displays an overview of the global fertility rate by country in 2020.

**Figure 5:**
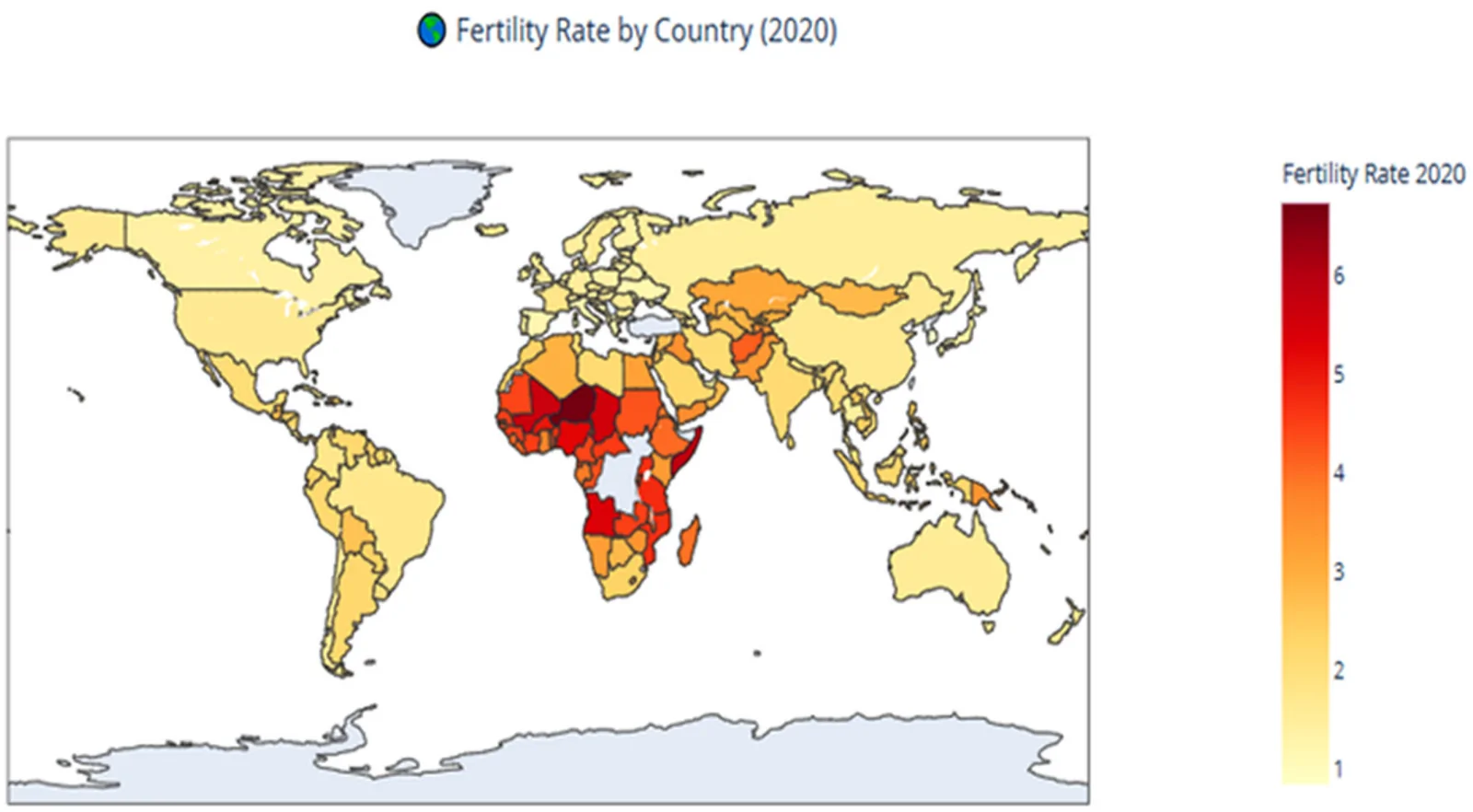
Global fertility rates overview by country in 2020 based on the Kaggle fertility rate dataset. The visualization presents the estimated number of births per couple using a color gradient.

Figure 5 shows an overview of global fertility rates by country for 2020. The color gradient illustrates the substantial geographical variation in fertility levels across countries. A lower fertility rate is indicated by a lighter shade of yellow, whereas a higher fertility rate is indicated by progressively darker shades of orange and red. Notable global fertility variations are also shown on the map. As the pale-yellow hues suggest, fertility rates are often lowest in countries located in sub-Saharan Africa, which, in contrast, have some of the world's highest fertility rates. Fertility rates are relatively high in parts of South Asia and the Middle East. These regions are represented by light red and orange colors. South America and Southeast Asia show a more moderate range of fertility rates. Therefore, our concentration of the highest fertility rates in specific regions highlights important demographic trends and potential implications for future population growth and development in those areas.

Later, a correlation heat map with data points indexed by year, spanning from 1960 to 2020, was visualized to understand how interrelationships changed over time, as displayed in Figure 6.

**Figure 6:**
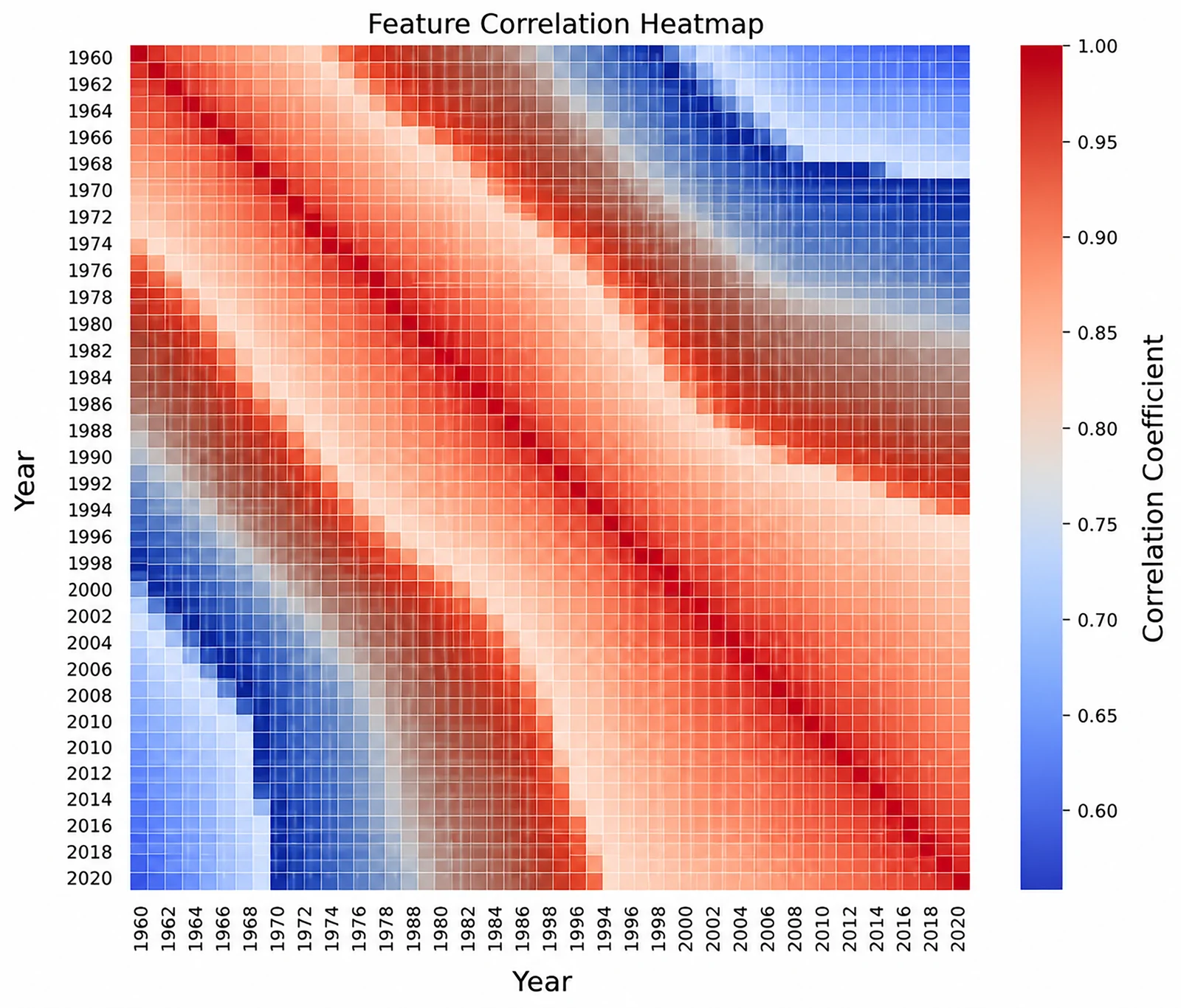
Feature correlation heatmap of fertility rate variables from 1960 to 2020. Each cell represents the linear correlation coefficient between fertility rates observed in two different years.

Figure 6 shows a heatmap that shows how different data points from 1960 to 2020 are related to each other in a straight line. Every cell in the heatmap denotes the Pearson’s correlation coefficient among data from the year indicated on the *y*-axis and the year indicated on the *x*-axis. Warmer colors (toward red) signify a strong positive correlation, indicating that features from these years tend to increase or decrease together. Cooler colors (toward blue) represent a negative correlation, suggesting that features from these years tend to change in the reverse direction. The concentration of color imitates the strength of correlation. The strong red line along the main diagonal and nearby diagonals shows a strong positive autocorrelation, meaning that data points from consecutive or close years are very closely related. The link becomes weaker and colors shift toward cooler tones as the distance between years increases from the diagonal. The temporal relationships within the dataset are highlighted by this pattern, which suggests that the linear influence on the present is stronger from the immediate past than from the distant past.

Next, to observe how fertility rates have been falling, they are compared among the most populous countries: India, the United States, China, and Nigeria, from 1960 to 2020. Figure 7 shows the visualization.

**Figure 7:**
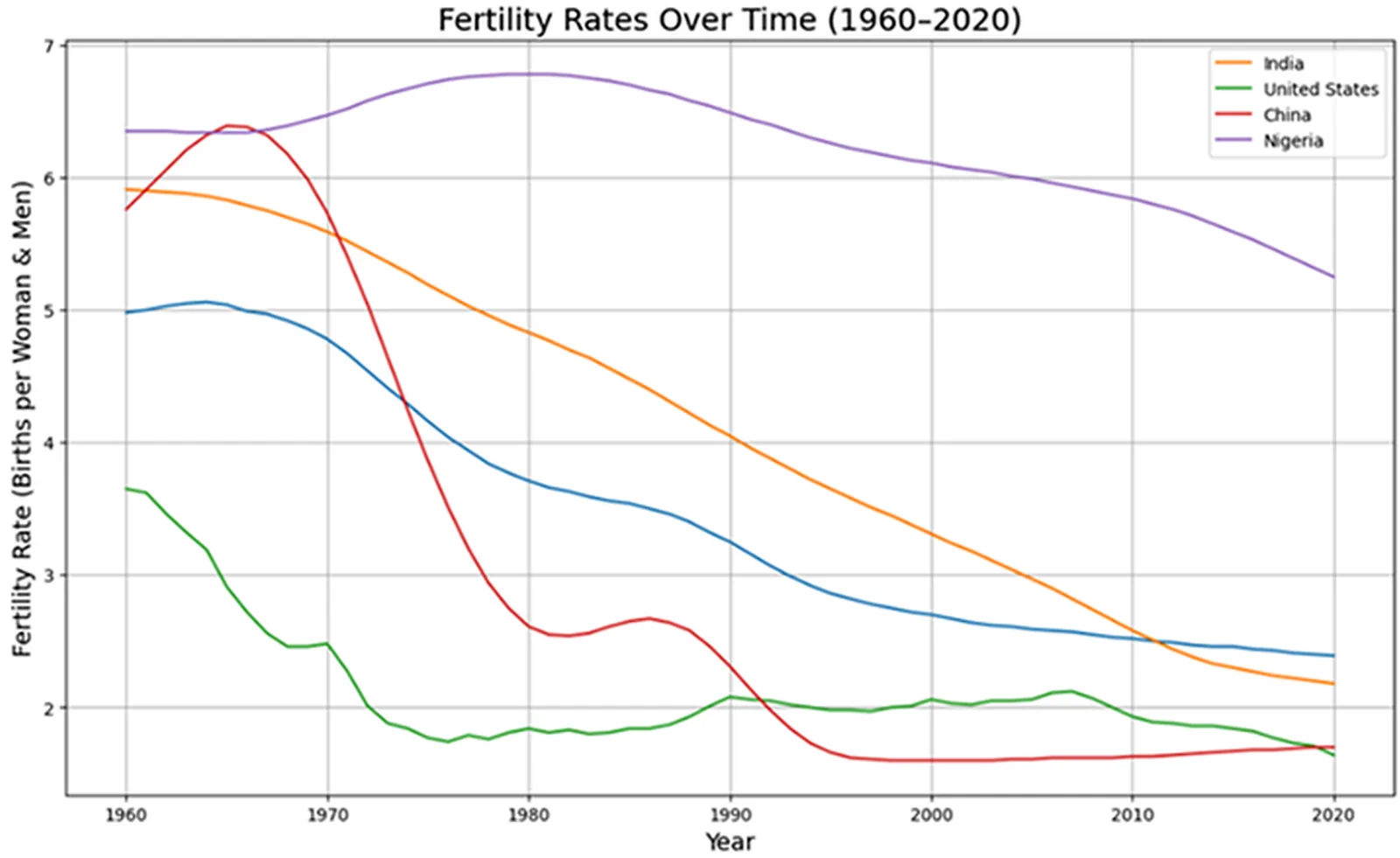
Fertility rate trends in selected countries from 1960 to 2020. The figure presents the temporal evaluation of the total fertility rates for India, the United States, China, and Nigeria over a 60-year period.

Figure 7 shows the fertility rate on the *y*-axis and the year on the *x*-axis. According to the legend, each colored line represents a different country. Different paths are shown for each country in the graph. Over the whole era, Nigeria maintained the highest fertility rate; however, it showed a slight dip in the later decades. In 1960, India's fertility rate was high, but it has been steadily declining since then. In comparison to Nigeria and India, the United States has a lower and more consistent fertility rate, which is trending downward with some fluctuations. The fertility rate in China shows more extreme swings: it spiked in the late 1960s and then dropped significantly, especially after population control measures were implemented. At the period's conclusion, China's fertility rate dropped to lowest of the four nations displayed.

Thus, the analysis of the fertility rate dataset revealed that globally, fertility rates across diverse nations show a drastic decrease in births, although this decline varies in magnitude and pattern owing to socioeconomic factors, government policies, cultural shifts, and health issues specific to each country. Therefore, there is a need to analyze and predict the causes of infertility in both men and women. To achieve this, the analysis focused on men’s fertility using EHR data.

### 4.5 Dataset 2: Fertility of men

First, a parallel coordinate was generated to visualize the relationships between the clinical and lifestyle features and their association with male fertility status. This visualization enables the simultaneous observation of multiple variables and facilitates the identification of patterns distinguish, as presented in Figure 8.

**Figure 8:**
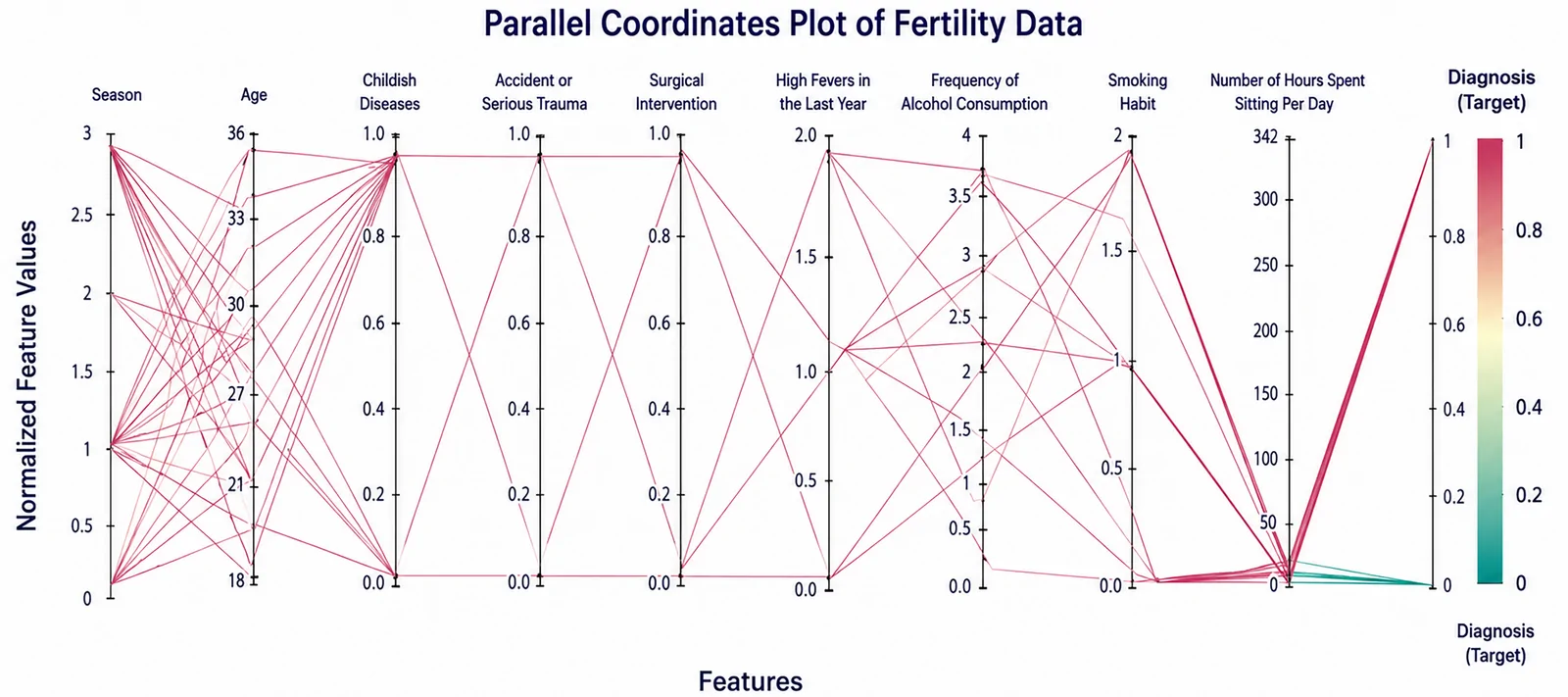
Predicted relationship between various features and the male fertility dataset. The color gradient indicates the fertility status, where 0 represents normal fertility and 1 represents altered fertility. This parallel coordinate analysis was employed to investigate the potential relationships, clustering patterns, and interactions among variables associated with male fertility outcomes.

As shown in Figure 8, each vertical axis represents a different feature: Season, Age, Childhood Diseases (0 = no and 1 = yes), Surgical Intervention (0 = no and 1 = yes), Accidents or Serious Trauma (0 = no and 1 = yes), and High Fevers in the Last Year (0 = no, 1= > 3 months, and 2 = more than 3 months), Frequency of Alcohol Consumption (categorical and numerically encoded), Smoking Habit (categorical and numerically encoded), Quantity of Hours Sitting per Day, and the diagnosis (0 = “Normal” and 1 = “Altered”). Each line traversing the parallel axes represents a single data point (an individual), and the color line indicates the fertility diagnosis, ranging from teal (0—“Normal”) to pink (1—“Altered”). Binary and categorical variables were converted into numerical labels using the label encoding method. However, continuous variables were normalized using Min-Max normalization before visualization. The plot shows that the two fertility classes overlap across most features, indicating that no single variable can distinguish the classes. Greater variation was observed for age, frequency of alcohol consumption, and number of hours spent sitting per day, whereas childhood diseases, accidents or serious trauma, and surgical intervention exhibited similar distributions in both classes. Class-wise statistics further indicate that the average age is 30.03 years for the normal class and 30.67 years for the altered class. These observations suggest that male fertility prediction depends on the combined influence of multiple demographic, lifestyle, and clinical factors.

Possible correlations and patterns between characteristics and diagnoses were identified by drawing lines across parallel axes. For example, lines with different colors (diagnosis) tend to cluster or separate along specific axes, which could indicate that these characteristics impact the differentiation between normal and disturbed fertility.

Ranges of features for every diagnosis: The feature ranges linked to different diagnosis colors are examined. Is there a correlation between, say, a certain age group or season and a higher concentration of people who exhibit “altered” fertility?

When examining correlations or anticorrelations, one should observe lines that consistently cross between two neighboring axes, which could indicate an inverted relationship, or lines that tend to maintain their relative order, which could indicate a positive association.

Assess the impact of categorical variables by examining the distribution of factors such as smoking habits and alcohol use over the two diagnostic colors.

In sum, the correlations between various variables in fertility data are more easily observed with the use of a parallel coordinates plot, which aids in the visual detection of potential connections for fertility diagnostics. A more detailed quantitative analysis is provided below.

In the next step, the dataset's male fertility diagnoses were forecasted to classify them according to counts that show how many people are impacted by infertility. Figure 9 depicts the distribution of male fertility diagnoses.

**Figure 9:**
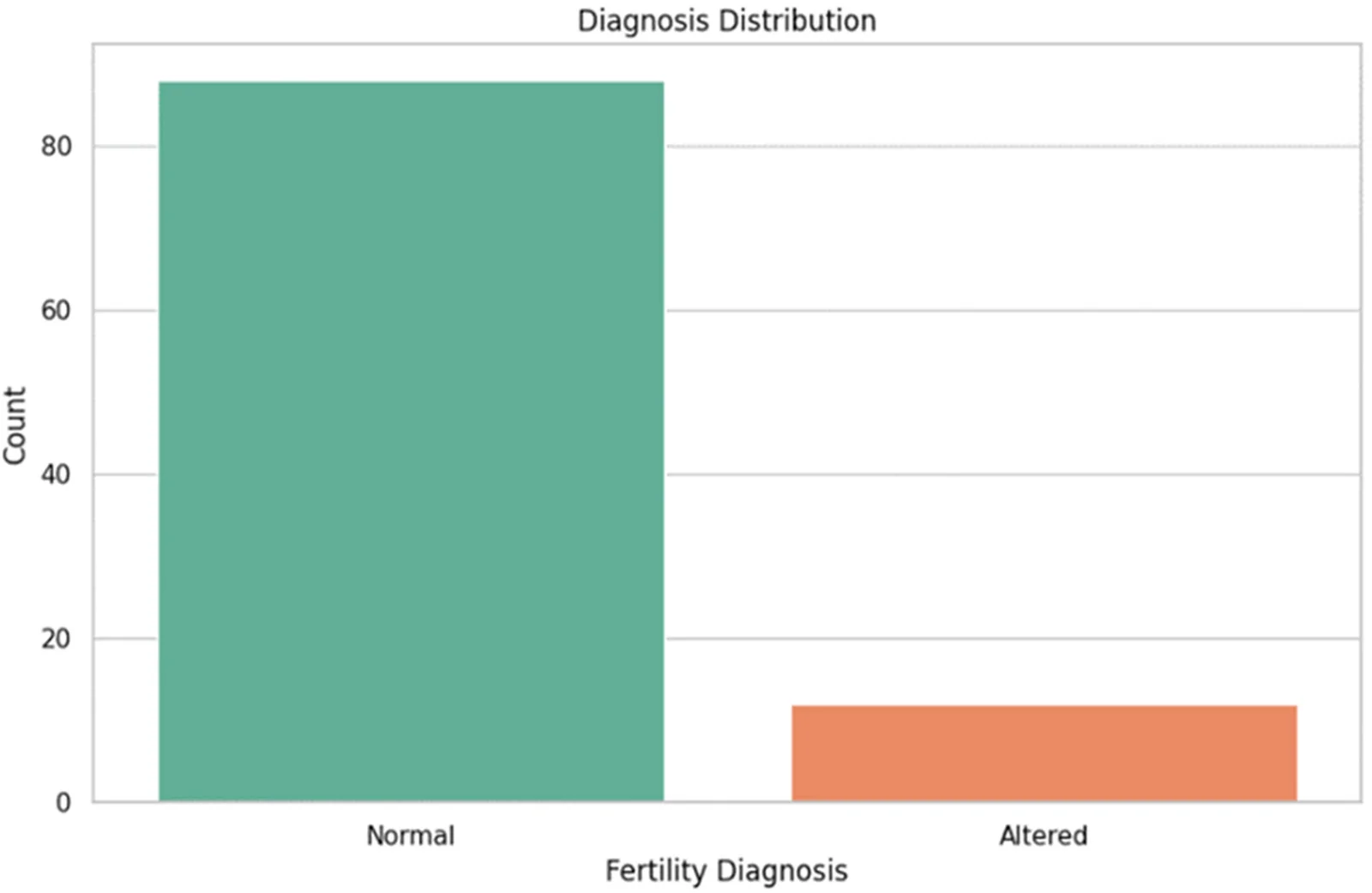
Male fertility diagnosis distribution. This class distribution analysis was performed to assess the dataset balance and identify potential class imbalance issues that could influence the predictive model performance.

In Figure 9, the *x*-axis shows two diagnostic categories, “Normal” and “Altered,” while the *y*-axis shows the frequency or count of each diagnosis. The number of people categorized under a particular fertility diagnosis is proportional to the height of that bar chart, whereas the “altered” diagnosis was given to a smaller percentage of this group.

Next, we determined the correlation between smoking and male infertility by predicting male fertility diagnoses based on smoking habits. Figure 10 illustrates the relationship between fertility diagnosis and smoking habits.

**Figure 10:**
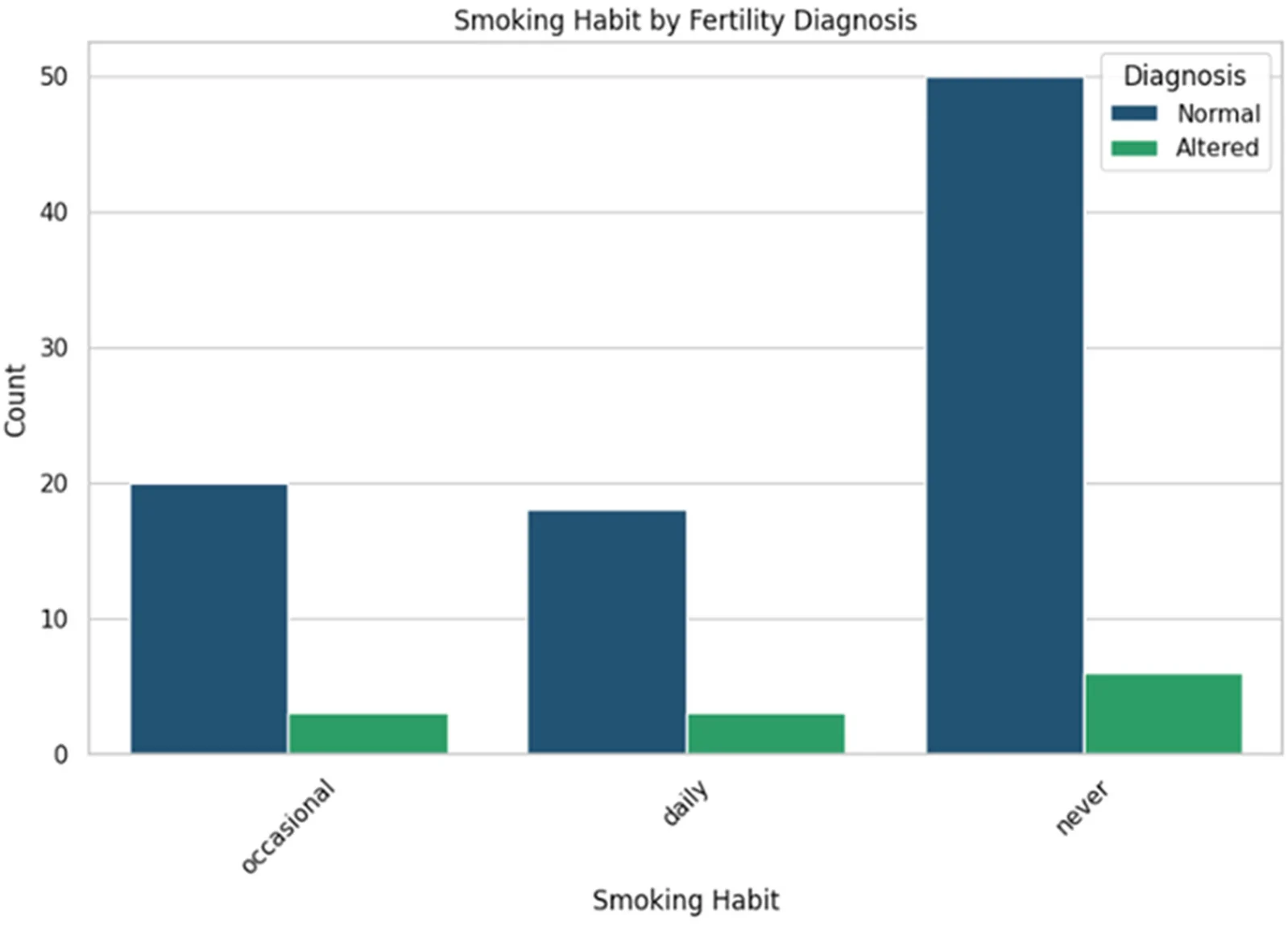
Fertility diagnosis and smoking habits relationship. The grouped bar chart was generated to investigate the association between the smoking behavior and fertility outcomes and to assess whether smoking frequency contributes to fertility classification.

In Figure 10, the *x*-axis depicts the frequency of smoking with three habits: “occasional,” “daily,” and “never.” The *y*-axis represents the frequency or quantity of each smoking habit category. The chart clearly displays the smoking habits of the two diagnostic groups. The “never smoked” category is largest in both groups, with the proportion of individuals who are “occasional” or “daily” smokers appearing relatively higher in the “normal” fertility diagnosis group compared to the “altered” fertility diagnosis group. Therefore, smoking habits did not exhibit a strong association with infertility within this dataset.

Then, fertility diagnosis was predicted based on four seasons: spring, fall, winter, and summer, because different seasons may also cause the males to be infertile. Figure 11 shows a visualization of this.

**Figure 11:**
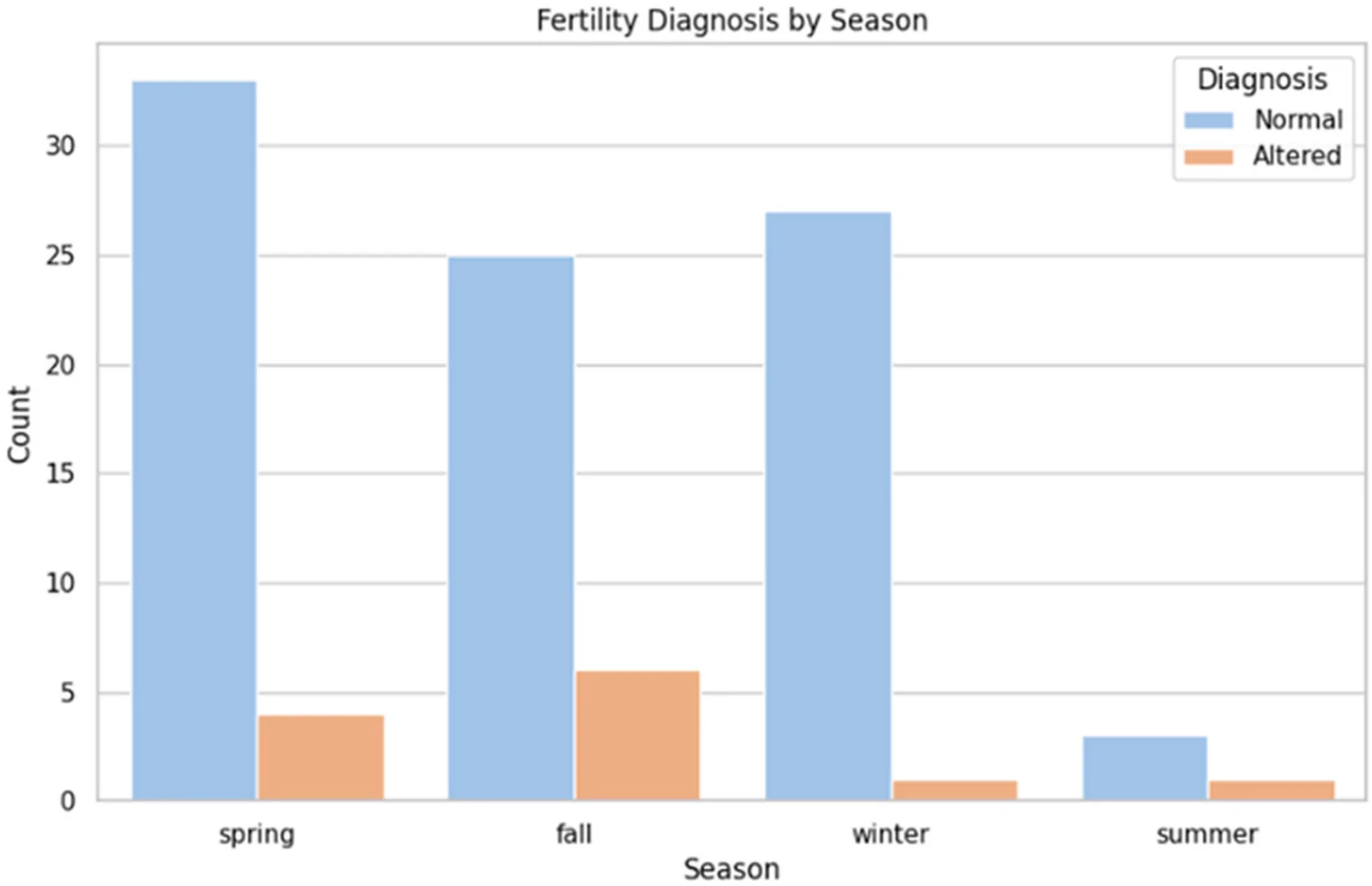
Fertility diagnosis by season in males. This seasonal analysis was performed to investigate the potential associations between seasonal variations and male fertility outcomes.

In Figure 11, the *x*-axis denotes different seasons, while the *y*-axis indicates the count or frequency of individuals within every season, further segmented by their fertility diagnosis. The light blue bars show people with a “normal” fertility diagnosis, and light orange bars show people with an “altered” fertility diagnosis. The chart reveals the number of normal and altered fertility diagnoses observed in each season. The “Normal” diagnosis consistently had a higher count than “Altered” diagnosis across all seasons. Specifically, the number of “normal” diagnoses was highest in spring, followed by fall and winter, and lowest in summer. The number of “altered” diagnoses was relatively low across all seasons. It was highest in fall, followed by spring, summer, and winter. Based on the findings from the Altered Diagnosis, seasonal variation exhibited an observable association with infertility outcomes in this dataset.

Later, fertility diagnosis was predicted based on the age and current lifestyle, as age plays a significant role in infertility issues among males. Figure 12 illustrates this visualization.

**Figure 12:**
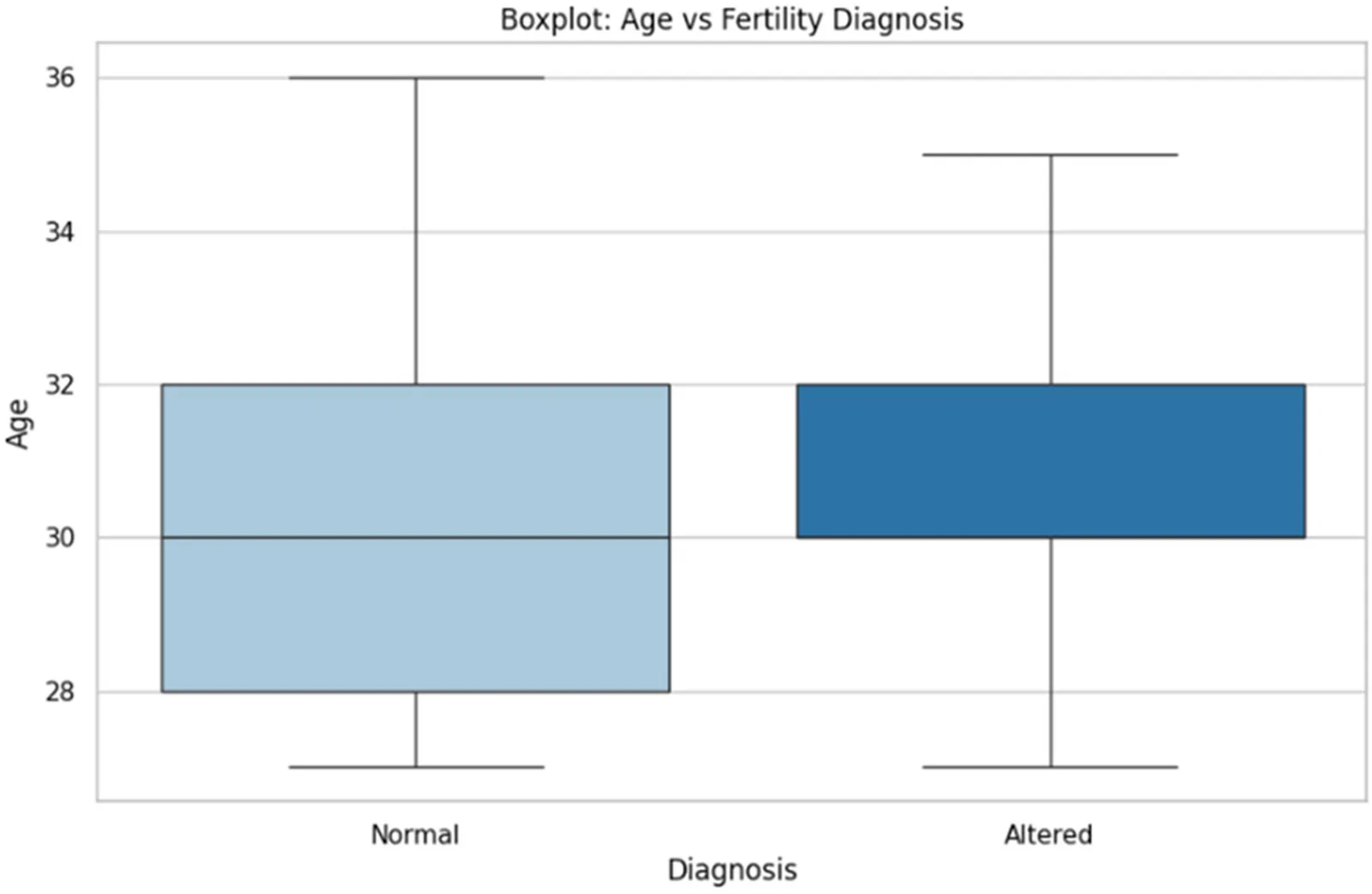
Fertility diagnosis based on age. This statistical visualization was generated to investigate the potential age-related differences associated with fertility outcomes.

In Figure 12, the *x*-axis denotes the diagnostic categories “Normal” and “Altered,” while the *y*-axis indicates the age of individuals. Each box plot visually summarizes the key statistical features of the age distribution for each diagnostic group. The central horizontal line within each box denotes the median age. The box itself involves the interquartile range (IQR), comprising the middle 50% of ages. The whiskers extending from the boxes indicate the typical range of data, usually defined as 1.5 times the IQR. Therefore, infertility affects some men after 30 years of age.

Then, the diagnosis of fertility issues in men was predicted based on high alcohol consumption. The predicted visualization is presented in Figure 13.

**Figure 13:**
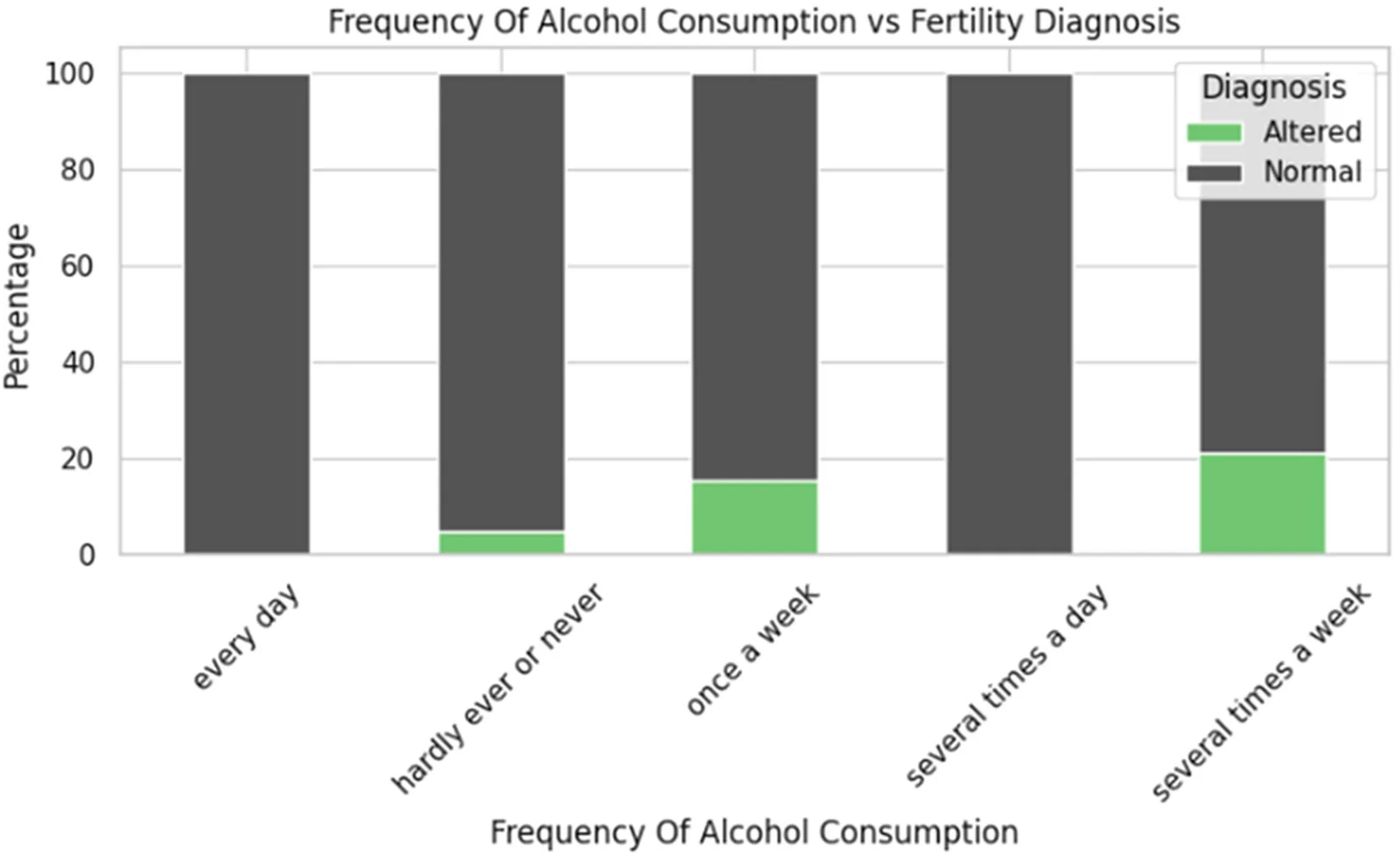
Frequency diagnosis based on alcohol consumption. A stacked bar chart was generated to evaluate the potential associations between alcohol consumption behavior and fertility outcomes and to examine whether the frequency of drinking contributes to fertility status classification.

In Figure 13, the *x*-axis represents different categories of alcohol consumption frequency: “hardly ever or never,” “every day,” “once a week,” “several times a week,” and “several times a day.” The *y*-axis displays percentage, totaling 100% for each category of alcohol consumption. Within each bar, the proportion of individuals with a “normal” fertility diagnosis is shown in dark gray, while the proportion with an “altered” fertility diagnosis is shown in light green. The distribution of fertility diagnoses across different frequencies of alcohol consumption is as follows:

For individuals who consume alcohol “every day,” approximately 77% had a fertility diagnosis of “normal”, and approximately 23% had a fertility diagnosis of “altered.”
The fertility diagnosis for individuals who “hardly ever or never” drink alcohol was “normal” for approximately 95% of population, while “altered” was the case for approximately 5%.
A “normal” fertility diagnosis was given to nearly 85% of those who drink alcohol “once a week,” whereas approximately 15% received an “altered” diagnosis.
Nearly all individuals in this dataset who reported drinking alcohol “several times a day” have a “normal” fertility diagnosis.
For individuals who consume alcohol “several times a week,” approximately 79% had a fertility diagnosis of “normal,” and approximately 21% had a fertility diagnosis of “altered.”
Overall, the potential trend shows that lower frequencies of alcohol consumption (“hardly ever or never”) are associated with a higher percentage of “normal” fertility diagnoses. Conversely, higher frequencies of alcohol consumption (“every day” and “several times a week”) appear to have a slightly higher proportion of “altered” fertility diagnoses. The “several times a day” category in this dataset only includes individuals with a “normal” diagnosis. Consequently, this suggests a possible association between the frequency of alcohol consumption and infertility in males.

## 5 Evaluation of model performance

Multiple classical LSTM architectures and a proposed Quantum LSTM model were evaluated on two datasets: (i) global fertility rate time-series data and (ii) her-derived male fertility data. The model performance was assessed using precision, accuracy, F1-score, recall, and computational time. To mitigate overfitting and sampling bias arising from limited data availability, three internal validation strategies were employed: fivefold cross-validation, repeated random train-test splits, and bootstrap resampling.

Among classical deep learning models, unilateral LSTM consistently achieved the strongest performance, yielding accuracies of 91.2% for the fertility rate dataset and 92.1% for the male fertility dataset, while exhibiting lower computational overhead than more complex LSTM variants. This suggests that for relatively small and structured datasets, simpler temporal architectures may generalize better than deeper or more expressive sequence models.

The proposed quantum LSTM achieved the highest predictive accuracy across both datasets: 92.6% for the fertility rate data and 93.8% for the male fertility data, with marginally reduced training time relative to classical LSTM models. Although these improvements were consistent across cross-validation and resampling procedures, the absolute performance gains over the best performing classical model were modest (approximately 1%–2%). To determine whether the experimental performance differences between Quantum LSTM and the strongest classical baseline were statistically significant, a Friedman test followed by pairwise Wilcoxon signed-rank assessments was applied across fivefold cross-validation, which confirmed that this difference was statistically significant (*P* = 0.031 and *P* = 0.024) with small to moderate effect sizes (Cohen's *d* = 0.42 and 0.48). These results indicate that Quantum LSTM consistently achieved the highest average rank; the observed improvements should be interpreted cautiously because of the limited sample size. These findings indicate that quantum-enhanced representations may provide incremental benefits in modeling complex, heterogeneous temporal patterns rather than a dramatic performance lead.

Bootstrap confidence interval analysis further showed that the quantum LSTM exhibited narrow confidence bounds, suggesting improved stability under repeated resampling. However, given the limited dataset size and simulator-based quantum execution, these results should be understood as preliminary suggestions of feasibility rather than conclusive proof of quantum advantage.

### 5.1 Interpretation of fertility rate analysis

The analysis of global fertility rate data from 1960 to 2020 confirms a well-established demographic trend: fertility rates have declined across most regions worldwide. Countries such as China and India have pronounced declines, likely reflecting socioeconomic development, public health interventions, and policy-driven population control measures. This component of the study serves primarily as contextual motivation, reinforcing the importance of investigating infertility at both the population and individual levels, rather than as a novel demographic discovery.

### 5.2 Insights from male fertility EHR data

Multivariate visualization and predictive modeling of the male fertility dataset revealed associations, not causal relationships, between fertility outcomes and several clinical and lifestyle factors. Age, frequency of alcohol consumption, seasonal variation, and selected medical history variables showed measurable contributions to the classification performance.

Smoking behavior and sedentary activity exhibited inconsistent or weak associations within this dataset, suggesting that their influence may be population-specific or confounded by unobserved variables. Importantly, the dataset size (*n* = 100) limits the statistical power, and the observed patterns would be interpreted as hypothesis-producing conclusive clinical evidence. These findings do not establish definitive clinical thresholds or diagnostic rules.

Overall, these results demonstrate that her-derived multivariate data, when combined with sequential deep learning models, can support exploratory risk stratification and pattern recognition in male infertility. However, they did not establish definitive clinical thresholds or diagnostic rules.

### 5.3 Limitations

This study has several limitations. First, the male fertility dataset was small (*n* = 100), which restricted the statistical power and limited generalizability, despite the use of multiple internal validation strategies. All deep learning models are at an elevated risk of overfitting on datasets of this size, despite regulatory measures. Second, model evaluation relied exclusively on internal validation, and no external datasets were available for independent verification of performance. Third, the quantum LSTM was executed using the PennyLane default simulator, which does not reflect real hardware constraints, such as gate noise, decoherence, or qubit connectivity limitations. Fourth, the performance improvements of the Quantum LSTM over classical models were modest and cannot be interpreted as evidence of a definitive quantum advantage. Fifth, all observed relationships between fertility outcomes and clinical or lifestyle variables were associative rather than causal. Finally, the dataset does not include genetic, hormonal, imaging, or longitudinal data, and the proposed models are not intended for direct clinical deployment. Future work will prioritize larger, multicenter EHR cohorts, validation on real quantum hardware, and integration of multimodal biological data to more rigorously assess the clinical utility of quantum-enhanced learning models in reproductive medicine.

## 6 Conclusion

Infertility represents a significant global health issue with substantial social and psychological implications. Advances in EHR systems have enabled a more comprehensive analysis of fertility-related data, offering opportunities to explore complex multivariate patterns that are difficult to capture using traditional analytical approaches. This study examined global fertility trends and her-derived male fertility data to evaluate classical and quantum-enhanced LSTM models under data-constrained conditions. Among the classical architectures, unilateral LSTM provided the most consistent and computationally efficient performance (91.2% and 92.1% accuracy on the fertility rate and male fertility datasets, respectively). The proposed quantum LSTM achieved the highest accuracy on both datasets (92.6% and 93.8%), representing a 1%–2% point improvement over the best classical baseline, with statistically significant but small to moderate effect sizes (Wilcoxon *P* < 0.05, Cohen’s *d* = 0.4–0.5). These results demonstrate the feasibility of embedding a variational quantum circuit directly within the LSTM input gating mechanisms for small, heterogeneous fertility datasets and establish a reproducible benchmarking model combining classical LSTM variants, a quantum-enhanced LSTM, and SHAP-based explainability for exploratory male infertility risk stratification, rather than establishing a definitive computational or clinical advantage. The observed associations between fertility outcomes and demographic or lifestyle factors were exploratory and non-causal. Given the limited dataset size, internal-only validation, and simulator-based quantum execution, these findings should not be understood as evidence of clinical or computational superiority, and the proposed models are not intended for direct clinical deployment. Future work with larger, multicenter EHR cohorts with externally validated datasets, real quantum hardware validation beyond simulator-based experiments, and integration of multimodal biological data, including genetic, hormonal, and imaging data, is required to assess generalizability and practical relevance.

## Data Availability

The original contributions presented in this study are included in the article/Supplementary Material, further inquiries can be directed to the corresponding author.
